# The Achilles’ heel of senescent cells: from transcriptome to senolytic drugs

**DOI:** 10.1111/acel.12344

**Published:** 2015-04-22

**Authors:** Yi Zhu, Tamara Tchkonia, Tamar Pirtskhalava, Adam C Gower, Husheng Ding, Nino Giorgadze, Allyson K Palmer, Yuji Ikeno, Gene B Hubbard, Marc Lenburg, Steven P O’Hara, Nicholas F LaRusso, Jordan D Miller, Carolyn M Roos, Grace C Verzosa, Nathan K LeBrasseur, Jonathan D Wren, Joshua N Farr, Sundeep Khosla, Michael B Stout, Sara J McGowan, Heike Fuhrmann-Stroissnigg, Aditi U Gurkar, Jing Zhao, Debora Colangelo, Akaitz Dorronsoro, Yuan Yuan Ling, Amira S Barghouthy, Diana C Navarro, Tokio Sano, Paul D Robbins, Laura J Niedernhofer, James L Kirkland

**Affiliations:** 1 Robert and Arlene Kogod Center on Aging, Mayo ClinicRochester, MN, USA; 2 Section of Computational Biomedicine, Boston University School of MedicineBoston, MA, USA; 3 Departments of Pathology, Barshop Institute for Longevity and Aging Studies, The University of Texas Health Science CenterSan Antonio, TX, USA; 4 Research Service, Geriatric Research and Education Clinical Center, Audie L. Murphy VA Hospital South Texas Veterans Health Care SystemSan Antonio, TX, USA; 5 Department of Biochemistry and Molecular Biology, Oklahoma Medical Research FoundationOklahoma City, OK, USA; 6 Department of Metabolism and Aging, The Scripps Research InstituteJupiter, FL, USA

**Keywords:** dasatinib, dependence receptors, ephrins, p21, PI3K delta, plasminogen-activated inhibitor, quercetin

## Abstract

The healthspan of mice is enhanced by killing senescent cells using a transgenic suicide gene. Achieving the same using small molecules would have a tremendous impact on quality of life and the burden of age-related chronic diseases. Here, we describe the rationale for identification and validation of a new class of drugs termed senolytics, which selectively kill senescent cells. By transcript analysis, we discovered increased expression of pro-survival networks in senescent cells, consistent with their established resistance to apoptosis. Using siRNA to silence expression of key nodes of this network, including ephrins (EFNB1 or 3), PI3Kδ, p21, BCL-xL, or plasminogen-activated inhibitor-2, killed senescent cells, but not proliferating or quiescent, differentiated cells. Drugs targeting these same factors selectively killed senescent cells. Dasatinib eliminated senescent human fat cell progenitors, while quercetin was more effective against senescent human endothelial cells and mouse BM-MSCs. The combination of dasatinib and quercetin was effective in eliminating senescent MEFs. *In vivo*, this combination reduced senescent cell burden in chronologically aged, radiation-exposed, and progeroid *Ercc1*^−/Δ^ mice. In old mice, cardiac function and carotid vascular reactivity were improved 5 days after a single dose. Following irradiation of one limb in mice, a single dose led to improved exercise capacity for at least 7 months following drug treatment. Periodic drug administration extended healthspan in *Ercc1*^−/Δ^ mice, delaying age-related symptoms and pathology, osteoporosis, and loss of intervertebral disk proteoglycans. These results demonstrate the feasibility of selectively ablating senescent cells and the efficacy of senolytics for alleviating symptoms of frailty and extending healthspan.

## Introduction

Aging is the leading risk factor for the chronic diseases that account for the bulk of morbidity, mortality, and health costs (Goldman *et al*., 2013). A fundamental aging mechanism that likely contributes to chronic diseases and age-related dysfunction is cellular senescence (Kirkland, 2013b,a; Tchkonia *et al*., 2013; Kirkland & Tchkonia, 2014). Senescence refers to the essentially irreversible growth arrest that occurs when cells are subjected to potentially oncogenic insults (Tchkonia *et al*., 2013). Even though senescent cell abundance in aging or diseased tissues is low, achieving a maximum of 15 percent of nucleated cells in very old primates, senescent cells can secrete pro-inflammatory cytokines, chemokines, and extracellular matrix proteases, which together constitute the senescence-associated secretory phenotype or SASP (Herbig *et al*., 2006; Coppé *et al*., 2008; Kuilman *et al*., 2008). The SASP likely contributes to the correlation between senescent cell accumulation and local and systemic dysfunction and disease. Consistent with a role for cellular senescence in causing age-related dysfunction, clearing senescent cells by activating a drug-inducible ‘suicide’ gene enhances healthspan and delays multiple age-related phenotypes in genetically modified progeroid mice (Baker *et al*., 2011). Interestingly, despite only clearing 30 percent of the senescent cells, improvement in age-related phenotypes is profound. Thus, interventions that reduce the burden of senescent cells could ameliorate age-related disabilities and chronic diseases as a group (Tchkonia *et al*., 2013; Kirkland & Tchkonia, 2014).

Despite their harsh internal and external microenvironments, senescent cells are viable. They survive even though they have active DNA damage responses, heightened metabolic flux, and increased local levels of SASP inflammatory cytokines and other factors that are able to induce apoptosis. Indeed, senescent cells are better able to withstand stresses such as serum deprivation than nonsenescent cells (Wang, 1995; Fridman & Lowe, 2003). *In vivo*, senescent cells appear to be removed by the immune system (Xue *et al*., 2007), rather than apoptosis or necrosis. Therefore, we hypothesized that (i) anti-apoptotic, pro-survival mechanisms could be up-regulated in senescent cells and (ii) interfering with these protective mechanisms might achieve selective elimination of senescent cells. Based on these hypotheses, here we identified several clinically used drugs that induce apoptosis preferentially of senescent cells *in vitro* and *in vivo*, leading to improved cardiovascular function and exercise endurance, reduced osteoporosis and frailty, and extended healthspan in several murine systems.

## Results

### The senescent transcriptome and anti-apoptotic pathways

**Fig 1:**
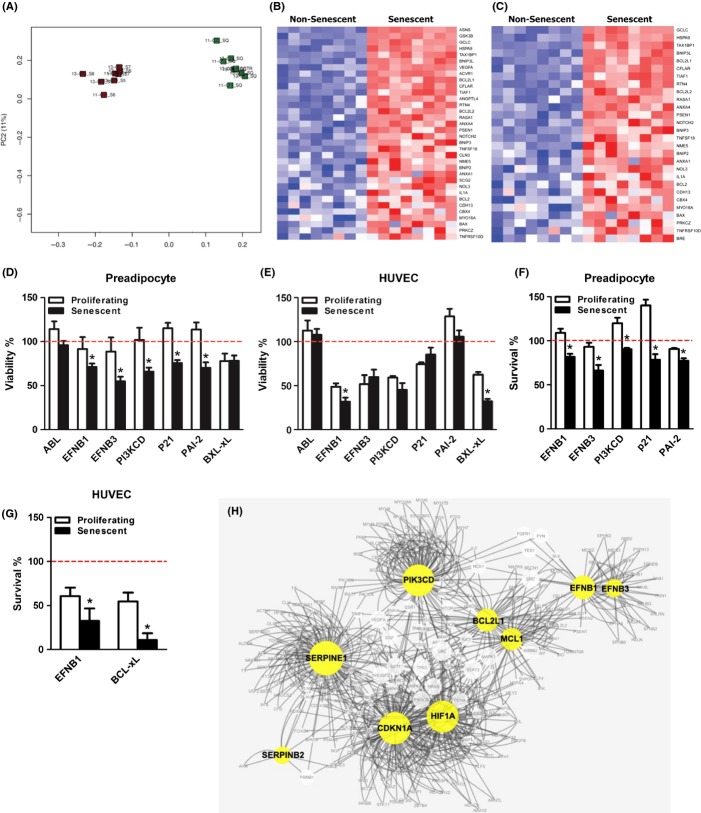
Senescent cells can be selectively targeted by suppressing pro-survival mechanisms. (A) Principal components analysis of detected features in senescent (green squares) vs. nonsenescent (red squares) human abdominal subcutaneous preadipocytes indicating major differences between senescent and nonsenescent preadipocytes in overall gene expression. Senescence had been induced by exposure to 10 Gy radiation (vs. sham radiation) 25 days before RNA isolation. Each square represents one subject (cell donor). (B, C) Anti-apoptotic, pro-survival pathways are up-regulated in senescent vs. nonsenescent cells. Heat maps of the leading edges of gene sets related to anti-apoptotic function, ‘negative regulation of apoptosis’ (B) and ‘anti-apoptosis’ (C), in senescent vs. nonsenescent preadipocytes are shown (red = higher; blue = lower). Each column represents one subject. Samples are ordered from left to right by proliferative state (*N* = 8). The rows represent expression of a single gene and are ordered from top to bottom by the absolute value of the Student *t* statistic computed between the senescent and proliferating cells (*i.e*., from greatest to least significance, see also Fig. S8). (D–E) Targeting survival pathways by siRNA reduces viability (ATPLite) of radiation-induced senescent human abdominal subcutaneous primary preadipocytes (D) and HUVECs (E) to a greater extent than nonsenescent sham-radiated proliferating cells. siRNA transduced on day 0 against ephrin ligand B1 (EFNB1), EFNB3, phosphatidylinositol-4,5-bisphosphate 3-kinase delta catalytic subunit (PI3KCD), cyclin-dependent kinase inhibitor 1A (p21), and plasminogen-activated inhibitor-2 (PAI-2) messages induced significant decreases in ATPLite-reactive senescent (solid bars) vs. proliferating (open bars) cells by day 4 (100, denoted by the red line, is control, scrambled siRNA). *N* = 6; *P < 0.05; *t*-tests. (F–G) Decreased survival (crystal violet stain intensity) in response to siRNAs in senescent vs. nonsenescent preadipocytes (F) and HUVECs (G). *N* = 5; *P < 0.05; *t*-tests. (H) Network analysis to test links among EFNB-1, EFNB-3, PI3KCD, p21 (CDKN1A), PAI-1 (SERPINE1), PAI-2 (SERPINB2), BCL-xL, and MCL-1.

We first tested our hypotheses by comparing gene expression in senescent vs. nonsenescent cells using transcript array analysis (Fig.1A–C). We focused on fat cell progenitors, or preadipocytes, as they are arguably one of the most abundant types of senescent cells in humans (Tchkonia *et al*., 2010). Cultures were split and senescence induced in half of the cells using 10 Gy of ionizing radiation. Twenty-five days later, gene expression was measured using Affymetrix arrays and differential expression patterns identified by principal component analysis (see Supporting materials and methods Data S1 for details). Overall patterns of gene expression differed substantially between senescent and nonsenescent preadipocytes isolated from the same subjects (Fig.1A). Analyses of gene categories indeed revealed up-regulation of negative regulators of apoptosis (Fig.1B) and anti-apoptotic gene sets (Fig.1C) in senescent compared to nonsenescent cells (see also Supporting information Fig. S8).

### Senolytic siRNAs

We next employed RNA interference to identify potential ‘senolytic’ targets. We used the following rationale for the selection of senescence-associated genes to target with siRNAs. (i) Senescent cells rely on anti-apoptotic, pro-survival defenses to a greater extent than nonsenescent cells. (ii) Senescent cells have much in common with cancer cells, such as active DNA damage responses (Ghosal & Chen, 2013), except senescent cells do not divide. Thus pro-survival pathways, which when inhibited drive cancer cell apoptosis, might be good targets as long as the pathway is not linked to cell proliferation. (iii) Similarly to cancer cells, senescent cells are metabolically active, with increased glycolysis (Dorr *et al*., 2013). Thus, senescent cells may be more dependent on pathways that protect against metabolically induced apoptosis than are nonsenescent cells. (iv) Dependence receptors promote apoptosis unless they are prevented from doing so by the presence of their ligands (Goldschneider & Mehlen, 2010). Thus, senescent cells may rely more on dependence receptor ligands than nonsenescent cells, as is the case in cancer cells (Goldschneider & Mehlen, 2010; Xi *et al*., 2012). Therefore, we targeted anti-apoptotic pathway elements by RNA interference, including the dependence receptor ligands and metabolic pro-survival transcripts identified in oncology. Importantly, targets identified by this rationale have the potential to yield senolytics that also will have antitumor effects.

Of the 39 transcripts selected for knockdown by siRNA transfection, at least 17 affected the viability of senescent cells more than the viability of nonsenescent cells (Supporting information Table S1). Of these, six triggered senescent cell death, but had little effect on proliferating, nonsenescent cells in two human cell types, preadipocytes (Fig.1D,F) and endothelial cells (Fig.1E,G). Interestingly, the senolytic transcripts included components of ephrin survival-regulating dependence receptor mechanisms (Goldschneider & Mehlen, 2010), ephrin ligand (EFN) B1, and EFNB3, as well as the cyclin-dependent kinase inhibitor 1A (p21), plasminogen-activated inhibitor-2 (PAI-2), the phosphatidylinositol-4,5-bisphosphate 3-kinase delta catalytic subunit (PI3KCD), a PI3K family member involved in regulating multiple cellular functions, including survival (Datta *et al*., 1999; Osaki *et al*., 2004), and BCL-xL, which regulates mitochondrial-dependent apoptosis and is the target of certain anticancer drugs (Minn *et al*., 1999; Leech *et al*., 2000).

Interfering with expression of EFNB1 or 3, PI3KCD, p21, BCL-xL, or PAI-2 significantly reduced the viability (ATPLite intensity; Fig.1D) and survival (crystal violet; Fig.1F and Fig. S6) of senescent but not proliferating human abdominal subcutaneous preadipocytes. Reducing EFNB2 or 4 or PI3K isoforms other than PI3KCD had less or no effect (Fig. S2C; Table S1). siRNA transfection efficiencies and extent of mRNA knockdown were similar in senescent and proliferating preadipocytes (Fig. S1). Results were confirmed using second, distinct siRNAs or by Western immunoanalysis (Fig. S2A, B, & F). While proliferating human umbilical vein cells (HUVECs) tended to be generally susceptible to siRNAs under the conditions used, senescent HUVECs were more susceptible to EFNB1 and BCL-xL siRNAs than nonsenescent cells (Fig.1E,G). EFNB1 or 3 and PI3KCD siRNAs also interfered with the viability of preadipocytes made senescent by serial subculturing compared to nonsenescent cells (Fig. S2D) and did not interfere with the viability of quiescent, differentiated preadipocytes (Fig. S2E). Results were confirmed using crystal violet to measure cell survival (Fig.1G; Fig. S6).

Based on potential associations among the genes targeted by senolytic siRNAs, we tested whether the gene products could be components of a common pro-survival signaling network (Fig.1H). Network analysis of these proteins using the STRING database suggested interaction of the anti-apoptotic proteins that we identified in siRNA assays.

### Candidate senolytic drugs *in vitro*

**Fig 2:**
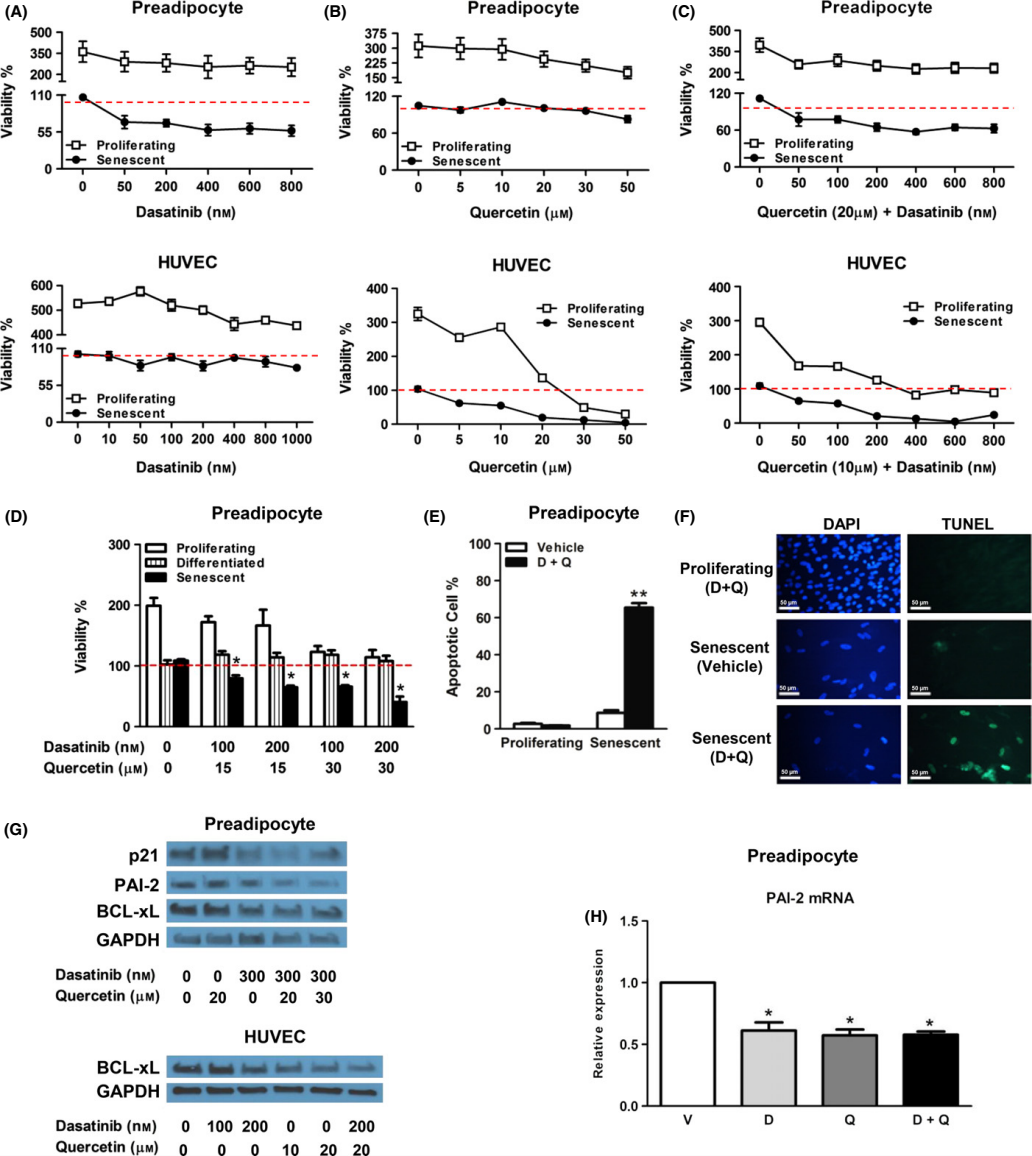
Dasatinib and quercetin target senescent cells. (A) D is more effective in selectively reducing viability (ATPLite) of senescent preadipocytes than HUVECs. Preadipocytes and HUVECs were exposed to different concentrations of D for 3 days. The red line denotes plating densities on day 0 of nondividing senescent (set to 100%) as well as proliferating nonsenescent cells (also set to 100%). Preadipocyte data are means ± SEM of four experiments in each of four different subjects. HUVEC data are means ± SEM of five replicates at each concentration. (B) Q is more effective in selectively reducing viability (ATPLite) of senescent HUVECs than preadipocytes. Proliferating and senescent preadipocytes and HUVECs were exposed to different concentrations of Q for 3 days. Preadipocyte data are means ± SEM of four experiments in each of four different subjects. HUVEC data are means ± SEM of five replicates at each concentration. (C) Combining D and Q selectively reduced viability of both senescent preadipocytes and senescent HUVECs. Proliferating and senescent preadipocytes and HUVECs were exposed to a fixed concentration of Q and different concentrations of D for 3 days. Optimal Q concentrations for inducing death of senescent preadipocyte and HUVEC cells were 20 and 10 μm, respectively. (D) D and Q do not affect the viability of quiescent fat cells. Nonsenescent preadipocytes (proliferating) as well as nonproliferating, nonsenescent differentiated fat cells prepared from preadipocytes (differentiated), as well as nonproliferating preadipocytes that had been exposed to 10 Gy radiation 25 days before to induce senescence (senescent) were treated with D+Q for 48 h. *N* = 6 preadipocyte cultures isolated from different subjects. *P < 0.05; anova. 100% indicates ATPLite intensity at day 0 for each cell type and the bars represent the ATPLite intensity after 72 h. The drugs resulted in lower ATPLite in proliferating cells than in vehicle-treated cells after 72 h, but ATPLite intensity did not fall below that at day 0. This is consistent with inhibition of proliferation, and not necessarily cell death. Fat cell ATPLite was not substantially affected by the drugs, consistent with lack of an effect of even high doses of D+Q on nonproliferating, differentiated cells. ATPLite was lower in senescent cells exposed to the drugs for 72 h than at plating on day 0. As senescent cells do not proliferate, this indicates that the drugs decrease senescent cell viability. (E, F) D and Q cause more apoptosis of senescent than nonsenescent primary human preadipocytes (terminal deoxynucleotidyl transferase dUTP nick end labeling [TUNEL] assay). (E) D (200 nM) plus Q (20 μm) resulted in 65% apoptotic cells (TUNEL assay) after 12 h in senescent but not proliferating, nonsenescent preadipocyte cultures. Cells were from three subjects; four replicates; **P < 0.0001; anova. (F) Primary human preadipocytes were stained with DAPI to show nuclei or analyzed by TUNEL to show apoptotic cells. Senescence was induced by 10 Gy radiation 25 days previously. Proliferating, nonsenescent cells were exposed to D+Q for 24 h, and senescent cells from the same subjects were exposed to vehicle or D+Q. D+Q induced apoptosis in senescent, but not nonsenescent, cells (compare the green in the upper to lower right panels). The bars indicate 50 μm. (G) Effect of vehicle, D, Q, or D+Q on nonsenescent preadipocyte and HUVEC p21, BCL-xL, and PAI-2 by Western immunoanalysis. (H) Effect of vehicle, D, Q, or D+Q on preadipocyte on PAI-2 mRNA by PCR. *N* = 3; *P < 0.05; anova.

We next tested whether drugs that target gene products that protect senescent cells from apoptosis are senolytic *in vitro*. Of 46 agents tested, dasatinib (D) and quercetin (Q) showed particular promise in clearing senescent cells. D is a inhibitor of multiple tyrosine kinases, used for treating cancers (Montero *et al*., 2011), and is known to interfere with EFNB-dependent supprepression of apoptosis (Chang *et al*., 2008; Xi *et al*., 2012). D preferentially reduced viability and caused cell death of senescent human preadipocytes, but was much less effective on senescent HUVECs (Fig.2A). Note that by day 3, proliferating preadipocytes increased by 2-5-fold in number vs. day 0 in the presence of D. The viability of nondividing, senescent preadipocytes from the same subjects decreased by 30–40% in the presence of 50 nm or greater D, indicating selective reduction in the viability of senescent cells. Q, a natural flavonol, inhibits PI3K, other kinases, and serpines (Olave *et al*., 2010; Bruning, 2013). In contrast to D, at low concentrations, Q reduced the viability and caused cell death of senescent HUVECs to a greater extent than proliferating cells, but was less effective on preadipocytes (Fig.2B). Note that at 10 μm Q, nonsenescent HUVECs achieved a 2-3-fold increase in cell number between days 0 and 3, while parallel cultures of senescent cells were reduced by 50%, indicating selective killing of senescent cells. The combination of D+Q afforded selective killing of both senescent preadipocytes and endothelial cells (Fig.2C-F). By day 3, the viability of nondividing senescent preadipocytes exposed to D+Q was reduced by ∼70% compared to day 0, while nonsenescent, proliferating cells had increased by 2 - 4-fold. By day 3, the viability of senescent HUVECs treated with 10 μm Q and 100 nm D was reduced by ∼50% compared to day 0. Parallel cultures of nonsenescent, proliferating HUVECs increased in number by 1.5-fold over the same period of time. This suggests that the combination of D+Q selectively targets a broader range of senescent cell types than either agent alone. In both senescent and nonsenescent cultured preadipocytes, D and Q reduced expression of the anti-apoptotic regulator PAI-2 (Fig.2G,H).

### Dasatinib and quercetin reduce senescent cells *in vivo*

**Fig 3:**
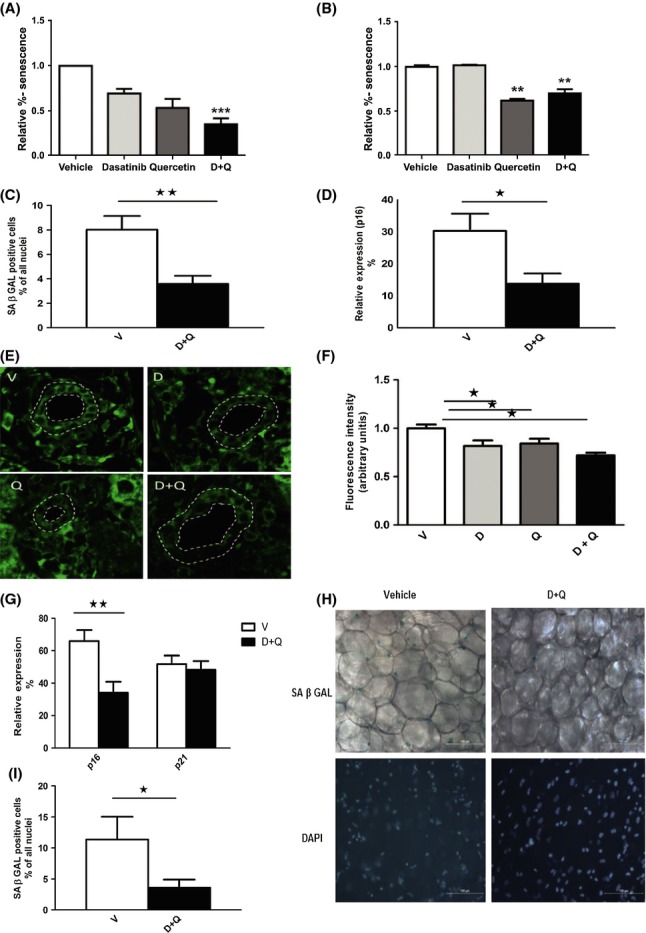
Dasatinib and quercetin reduce senescent cell abundance in mice. (A) Effect of D (250 nm), Q (50 μm), or D+Q on levels of senescent *Ercc1*-deficient murine embryonic fibroblasts (MEFs). Cells were exposed to drugs for 48 h prior to analysis of SA-βGal^+^ cells using C_12_FDG. The data shown are means ± SEM of three replicates, ***P < 0.005; *t*-test. (B) Effect of D (500 nM), Q (100 μm), and D+Q on senescent bone marrow-derived mesenchymal stem cells (BM-MSCs) from progeroid *Ercc1*^−/Δ^ mice. The senescent MSCs were exposed to the drugs for 48 h prior to analysis of SA-βGal activity. The data shown are means ± SEM of three replicates. **P < 0.001; anova. (C–D) The senescence markers, SA-βGal and p16, are reduced in inguinal fat of 24-month-old mice treated with a single dose of senolytics (D+Q) compared to vehicle only (V). Cellular SA-βGal activity assays and p16 expression by RT–PCR were carried out 5 days after treatment. *N* = 14; means ± SEM. **P < 0.002 for SA-βGal, *P < 0.01 for p16 (*t*-tests). (E–F) D+Q-treated mice have fewer liver p16^+^ cells than vehicle-treated mice. (E) Representative images of p16 mRNA FISH. Cholangiocytes are located between the white dotted lines that indicate the luminal and outer borders of bile canaliculi. (F) Semi-quantitative analysis of fluorescence intensity demonstrates decreased cholangiocyte p16 in drug-treated animals compared to vehicle. *N* = 8 animals per group. *P < 0.05; Mann–Whitney U-test. (G–I) Senolytic agents decrease p16 expression in quadricep muscles (G) and cellular SA-βGal in inguinal fat (H–I) of radiation-exposed mice. Mice with one leg exposed to 10 Gy radiation 3 months previously developed gray hair (Fig.5A) and senescent cell accumulation in the radiated leg. Mice were treated once with D+Q (solid bars) or vehicle (open bars). After 5 days, cellular SA-βGal activity and p16 mRNA were assayed in the radiated leg. *N* = 8; means ± SEM, p16: **P < 0.005; SA β-Gal: *P < 0.02; *t*-tests.

In anticipation of testing D+Q in a preclinical model, the drugs were tested for the efficacy in reducing the viability of senescent murine cells. The combination of D+Q led to a significant reduction in the number of senescent, C_12_FDG-positive, primary mouse embryonic fibroblasts (MEFs) compared to either drug alone (Fig.3A). Likewise, Q alone or D+Q caused a significant reduction in the number of senescent bone marrow-derived murine mesenchymal stem cells (Fig.3B). These data and those in Fig.2 demonstrate that both D and Q are able to selectively kill senescent cells in two species, albeit with distinct cell-type specificity. We tested whether D+Q administered by oral gavage was senolytic *in vivo*. We initially tested D+Q in old mice (> 24 months-old), as senescent cell burden increases in fat tissue with aging and both preadipocytes and endothelial cells contribute to senescent cell burden with aging in fat (Tchkonia *et al*., 2010). A single dose of D+Q (D: 5 mg kg^−1^ body weight and Q: 50 mg kg^−1^ by oral gavage here and in the following studies), a drug ratio that was most effective in senescent MEFs (data not shown), reduced SA-βgal^+^ cells (Fig.3C) and p16 mRNA (Fig.3D) in fat from old mice within 5 days. D+Q also reduced p16-positive cells in liver from old mice (Fig.3E-F). As with AP20187, the drug that activates selective killing of cells expressing p16 in transgenic *INK-ATTAC* mice (Baker *et al*., 2011), not all senescent cells were removed by D+Q., yet functional improvement was seen (see below) Following irradiation of one leg of wild-type mice, a single treatment with D+Q reduced p16 expression in muscle (Fig.3G) and SA-βGal^+^ cells in fat (Fig.3H,I) at the site of localized ionizing radiation exposure in these mice.

### Effects of senolytic agents on cardiovascular function in old mice

Cellular senescence is associated with cardiovascular dysfunction in humans (Tchkonia *et al*., 2013; Kirkland & Tchkonia, 2014), a major cause of morbidity and mortality in the elderly. While only mild cardiac dysfunction has been reported in old mice (Dai *et al*., 2009; Roos *et al*., 2013), substantial impairment in vascular reactivity is observed in aged mice (Roos *et al*., 2013). We tested whether treating 24-month-old mice with D+Q would improve cardiac ejection fraction (the fraction of heart volume pumped during each heart contraction) and vascular responses to acetylcholine, nitroprusside, or U46619 [endothelium-dependent relaxation, smooth muscle vascular reactivity to nitric oxide, and smooth muscle contractile function, respectively (Roos *et al*., 2013)]. To allow time for senescent cells to apoptose and exclude potential ‘off-target’ effects of the drugs on nonsenescent cell types, which require continued presence of the drugs, for example, through direct vasoactive/antioxidant effects or through changing NAD^+^ (Chen & Pace-Asciak, 1996; Ajay *et al*., 2006), we gave a single dose of the drugs and waited 5 days before assaying cardiac function. D and Q are cleared within 48 h of the last dose (Luo *et al*., 2006; Piantelli *et al*., 2006).

**Fig 4:**
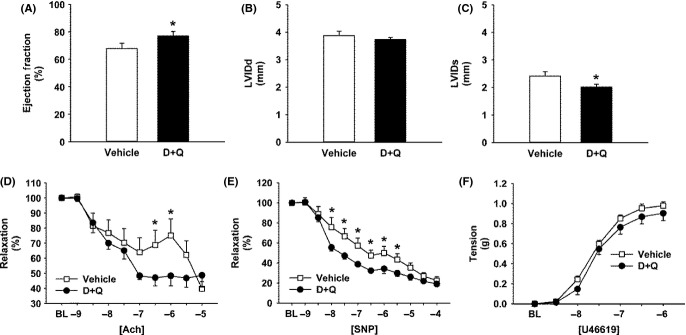
Effects of senolytic agents on cardiac (A–C) and vasomotor (D–F) function. D+Q significantly improved left ventricular ejection fraction of 24-month-old mice (A). Improved systolic function did not occur due to increases in cardiac preload (B), but was instead a result of a reduction in end-systolic dimensions (C; Table S3). D+Q resulted in modest improvement in endothelium-dependent relaxation elicited by acetylcholine (D), but profoundly improved vascular smooth muscle cell relaxation in response to nitroprusside (E). Contractile responses to U46619 (F) were not significantly altered by D+Q. In panels D–E, relaxation is expressed as the percentage of the preconstricted baseline value. Thus, for panels D–F, *lower* values indicate improved vasomotor function. *N* = 8 male mice per group. *P < 0.05; A–C: *t*-tests; D–F: anova.

Despite the fact that mice are relatively resistant to the development of age-related systolic dysfunction, treatment of 24-month-old mice with a single dose of D+Q significantly improved left ventricular ejection fraction (Fig.4A) and fractional shortening (Table S3), effects that were mediated by reductions in end-systolic cardiac dimensions (Fig.4C) but not cardiac preload (Fig.4B) or alterations in cardiac mass (Table S3). Consistent with previous reports from our group and others (Roos *et al*., 2013; Gioscia-Ryan *et al*., 2014), carotid arteries from aged mice displayed markedly impaired vascular relaxation in response to endothelium-dependent and -independent vasodilators compared to young mice. While D+Q elicited somewhat variable but statistically significant improvements in vascular endothelial function (Fig.4D), a complex amalgam of nitric oxide, endothelium-derived hyperpolarizing factors, and other vasoactive substances (Feletou & Vanhoutte, 2006), D+Q, yielded physiologically important and consistent improvements in vascular smooth muscle sensitivity to nitroprusside (Fig.4E). Interestingly, senescent cell clearance did not alter smooth muscle contractile function (Fig.4F). Collectively, these data suggest that senescent cells likely exert deleterious effects on cardiovascular function with chronological aging and that acute clearance of senescent cells may be a novel therapeutic approach to improve cardiovascular function and reduce morbidity and mortality from cardiovascular disease in the elderly.

### Effects on treadmill exercise capacity in mice after single leg radiation exposure

**Fig 5:**
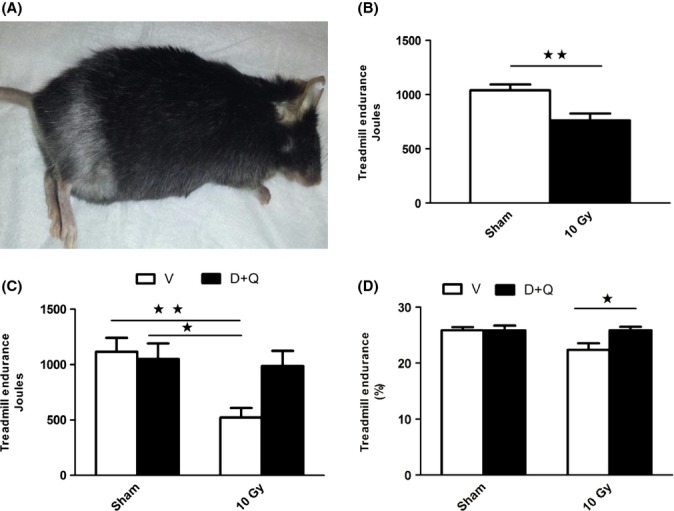
Senolytic administration alleviates radiation-induced impairment in treadmill exercise endurance. (A–B) One leg of 4-month-old mice was radiated at 10 Gy. Three months later, hair on the irradiated leg had turned gray (A) and treadmill exercise capacity (B) was lower in irradiated (*N* = 13) than sham-irradiated mice (*N* = 14). **P < 0.002; *t*-test. (C) Five days after a single dose of D+Q, treadmill endurance was better than in vehicle-treated controls. D+Q had no effect in sham-irradiated controls. (*N = 6-9* animals per group). Bars represent means ± SEM; *P < 0.05; **P < 0.001; anova; Tukey–Kramer test. (D) 7 months after a single dose of D+Q, treadmill endurance was again assayed. All groups ran on the treadmill on four occasions, each 1 week apart. Bars represent means ±SEM of the average performance of each group on each of the four occasions they ran. Endurance is shown as a function of the overall performance of all four groups on each occasion when mice ran (expressed as %: mean Joules per group/total Joules per all groups that day). * Different from the other groups; *P *< 0.05; anova; Duncan’s test.

To test further the hypothesis that D+Q functions through elimination of senescent cells, we tested the effect of a single treatment in a mouse leg irradiation model. One leg of 4-month-old male mice was irradiated at 10 Gy with the rest of the body shielded. Controls were sham-irradiated. By 12 weeks, hair on the irradiated leg turned gray (Fig.5A) and the animals exhibited reduced treadmill exercise capacity (Fig.5B). Five days after a single dose of D+Q, exercise time, distance, and total work performed to exhaustion on the treadmill was greater in the mice treated with D+Q compared to vehicle (Fig.5C). Senescent markers were reduced in muscle and inguinal fat 5 days after treatment (Fig.3G-I). At 7 months after the single treatment, exercise capacity was significantly better in the mice that had been irradiated and received the single dose of D+Q than in vehicle-treated controls (Fig.5D). D+Q-treated animals had endurance essentially identical to that of sham-irradiated controls. The single dose of D+Q had no effect on endurance 7 months later in sham-irradiated controls vs. vehicle. Thus, a single dose of D+Q 7 months previously led to sustained improvement in function, consistent with the ability of D+Q to clear damaged senescent cells acutely, resulting in enhanced later-life physical endurance.

### Extension of healthspan by periodic treatment of progeroid *Ercc1*^−/Δ^ mice with senolytics

**Fig 6:**
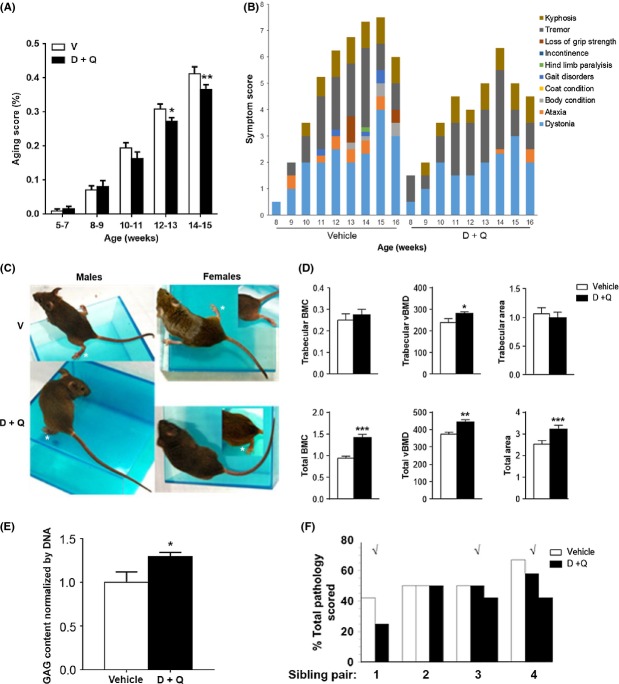
Periodic treatment with D+Q extends the healthspan of progeroid *Ercc1*^−/Δ^ mice. Animals were treated with D+Q or vehicle weekly. Symptoms associated with aging were measured biweekly. Animals were euthanized after 10–12 weeks. *N* = 7–8 mice per group. (A) Histogram of the aging score, which reflects the average percent of the maximal symptom score (a composite of the appearance and severity of all symptoms measured at each time point) for each treatment group and is a reflection of healthspan (Tilstra *et al*., 2012). *P < 0.05 and **P < 0.01 Student’s *t*-test. (B) Representative graph of the age at onset of all symptoms measured in a sex-matched sibling pair of *Ercc1*^−/Δ^ mice. Each color represents a different symptom. The height of the bar indicates the severity of the symptom at a particular age. The composite height of the bar is an indication of the animals’ overall health (lower bar better health). Mice treated with D+Q had delay in onset of symptoms (*e.g*., ataxia, orange) and attenuated expression of symptoms (*e.g*., dystonia, light blue). Additional pairwise analyses are found in Fig. S11. (C) Representative images of *Ercc1*^−/Δ^ mice from the D+Q treatment group or vehicle only. Splayed feet are an indication of dystonia and ataxia. Animals treated with D+Q had improved motor coordination. Additional images illustrating the animals’ gait and body condition are in Fig. S10. (D) Quantitative computed tomography (QCT)-derived bone parameters at the lumbar spine of 16-week-old *Ercc1*^−/Δ^ mice treated with either vehicle (*N* = 7) or drug (*N* = 8). BMC = bone mineral content; vBMD = volumetric bone mineral density. *P < 0.05; **P < 0.01; ***P < 0.001. (E) Glycosaminoglycan (GAG) content of the nucleus pulposus (NP) of the intervertebral disk. GAG content of the NP declines with mammalian aging, leading to lower back pain and reduced height. D+Q significantly improves GAG levels in *Ercc1*^*−/Δ*^ mice compared to animals receiving vehicle only. *P < 0.05, Student’s *t*-test. (F) Histopathology in *Ercc1*^*−/Δ*^ mice treated with D+Q. Liver, kidney, and femoral bone marrow hematoxylin and eosin-stained sections were scored for severity of age-related pathology typical of the *Ercc1*^*−/Δ*^ mice. Age-related pathology was scored from 0 to 4. Sample images of the pathology are provided in Fig. S13. Plotted is the percent of total pathology scored (maximal score of 12: 3 tissues x range of severity 0–4) for individual animals from all sibling groups. Each cluster of bars is a sibling group. White bars represent animals treated with vehicle. Black bars represent siblings that were treated with D+Q. The √ denotes the sibling groups in which the greatest differences in premortem aging phenotypes were noted, demonstrating a strong correlation between the pre- and postmortem analysis of frailty.

To demonstrate that treatment with D+Q can extend healthspan, we used the *Ercc1*^−/Δ^ mouse model of accelerated aging. These mice, which model human XFE progeria, have features resembling accelerated aging with a lifespan of 6 months (Dolle *et al*., 2011) and spontaneously develop progressive age-related chronic degenerative diseases (Gregg *et al*., 2011). MEFs deficient in ERCC1 or bone marrow-derived MSCs from *Ercc1*^*−/Δ*^ mice have increased senescence and are more susceptible to elimination by D+Q (Fig.3A,B). *Ercc1*^−/Δ^ mice were treated with 5 mg kg^−1^ D plus 50 mg kg^−1^ Q weekly by oral gavage or vehicle only (10% PEG400 in water) beginning at 4–6 weeks of age. Symptoms associated with aging were measured biweekly by an investigator blinded as to the treatment groups. Animals were euthanized after 10–12 weeks of treatment and molecular and histopathological endpoints measured. D+Q resulted in reduced expression of senescence markers in several tissues (Fig. S9and S12). This correlated with a significant reduction in a composite score of age-related symptoms (Fig.6A,B), including kyphosis, dystonia, tremors, loss of grip strength, coat condition, ataxia, urinary incontinence, impaired gait, hind limb paralysis, and poor body condition (Fig. S10). This reduction in symptoms indicates an extension of healthspan due to both the delay in onset of symptoms and attenuation of their severity (Fig.6B). In particular, the mice showed reduced dystonia and delayed onset of ataxia and gait disorders (Fig.6C and Fig. S10). In addition, quantitative computed tomography (pQCT) of lumbar spine demonstrated improved bone parameters in 16-week-old *Ercc1*^*−/Δ*^ mice treated with D+Q compared to animals treated with vehicle only (Fig.6D). Similarly, the level of proteoglycans in the nucleus pulposus of the intervertebral disk, a marker of age-related disk degeneration, was significantly increased in mice treated with D+Q, suggesting that treatment with D+Q can slow age-related dysfunction even of a relatively avascular tissue (Fig.6E). Finally, sections of liver, kidney, and the femoral bone space were stained with H&E and scored for age-related pathology by two pathologists blinded to the treatment groups. Composite pathology scores for sibling groups revealed reduced pathology in most animals treated with D+Q compared to siblings treated with vehicle only (Fig.6F). Remarkably, the sibling groups identified as having the most dramatic differences in pathology were identical to those identified as having the greatest difference in aging score (Fig.6B and Fig. S11), demonstrating a close correlation between pre- and postmortem endpoints. Taken together, these data demonstrate that periodic treatment with senolytics is sufficient to reduce the burden of senescence markers, reduce frailty, and extend healthspan significantly.

## Discussion

We previously demonstrated that the healthspan of transgenic mice can be enhanced by selectively killing senescent cells using a transgenic suicide gene (Baker *et al*., 2011). The identification of approaches to remove damaged, senescent cells would have a tremendous impact on quality of life and burden of age-related chronic diseases. To identify agents able to kill senescent cells, we hypothesized that senescent cells, like cancer cells, are dependent on anti-apoptotic pathways to ensure their survival following stress and damage. Based on this hypothesis, here we demonstrate that senescent cells indeed are susceptible to selective clearance by targeting pro-survival mechanisms using siRNAs and drugs, even at doses insufficient to kill normal proliferating or differentiated, quiescent cells. This observation opens up new approaches to develop clinically relevant small molecules or biologics that selectively eliminate senescent cells from nongenetically modified individuals, acting as senolytic agents. The prototype senolytic agents identified here, dasatinib and quercetin, have the ability to alleviate multiple aging phenotypes, as would be predicted if they truly act by eliminating senescent cells (Kirkland, 2013a; Kirkland & Tchkonia, 2014).

Interfering with expression of the ephrin dependence receptor ligands, EFNB1 or EFNB3, induces selective loss of senescent cells. Ephrin receptors are the largest family of receptor tyrosine kinases (Boyd *et al*., 2014). Together with ephrin ligands, these receptors coordinate tissue and organ patterning, cell positioning, and cell survival during development and tissue turnover in a cell type-specific manner (Xi *et al*., 2012). Ephrin B ligands, which span cell membranes, can act as both ligands and receptors, making contact with ephrin receptors on adjacent cells. EFNB ligands can participate in dependence networks that constrain both the cells they are located on and adjacent cells from undergoing apoptosis (Furne *et al*., 2009). Ephrin signaling has been linked to cellular senescence: EFNB3 overexpression can induce p21, PAI-1, the SASP, and SA-βGal activity during wound healing (Hafner *et al*., 2005). Interfering with expression of EFNB3 in cancer cells can induce apoptosis and disrupt pro-survival networks (Stahl *et al*., 2013). Similar to cancer cells, here we demonstrated that silencing EFNB ligands induces apoptosis selectively in senescent as compared to nonsenescent cells.

Silencing EFNB3 expression down-regulates AKT in cancer cells (Stahl *et al*., 2013). AKT is involved in regulating FOXO1 and mTOR, among other key pro-survival and metabolic homeostasis mechanisms (Chandarlapaty, 2012). PI3K is upstream of AKT, and the PI3KCD (catalytic subunit δ) is specifically implicated in the resistance of cancer cells to apoptosis. PI3KCD inhibition leads to selective apoptosis of cancer cells (Cui *et al*., 2012; Xing & Hogge, 2013). Consistent with these observations, we demonstrate that siRNA knockdown of the PI3KCD isoform, but not other PI3K isoforms, is senolytic in preadipocytes (Table S1).

p21 and PAI-1, both regulated by p53, are implicated in protection of cancer and other cell types from apoptosis (Gartel & Radhakrishnan, 2005; Kortlever *et al*., 2006; Schneider *et al*., 2008; Vousden & Prives, 2009). We found that p21 siRNA is senolytic (Fig.1D+F), and PAI-1 siRNA and the PAI-1 inhibitor, tiplaxtinin, also may have some senolytic activity (Fig. S3). We found that siRNA against another serine protease inhibitor (serpine), PAI-2, is senolytic (Fig.1D+F). Like PAI-1, PAI-2 can protect against apoptosis (Tonnetti *et al*., 2008; Delhase *et al*., 2012). EFNB1 and 3 expression appears linked to that of BCL-xL, PI3KCD, p21, PAI-1, and, indirectly, PAI-2, in published reports and by bioinformatics analysis (Fig.1H) (Hafner *et al*., 2005), suggesting these transcripts may be networked, a hypothesis that merits further study. Consistent with this hypothesis are our findings that D alone reduced the level of p21 protein, and furthermore, D plus Q reduced p21, PAI-2, and BCL-xL (Fig.2G,H).

D and Q are both approved for use in humans and appear to be relatively safe. Interestingly, imatinib, which is very closely related to D, was not senolytic, at least in preadipocytes (Fig. S4). D and Q are promiscuous, like many drugs that affect signaling pathway kinases either directly or indirectly. Despite this, they appear to have more senolytic activity against some types of senescent cells than others, and overall they appear to work better in combination than individually. Thus, one strategy to follow in developing future senolytic agents will be to use promiscuous agents or combinations of drugs to target anti-apoptotic networks. Alternatively, additional senolytic agents could be developed by de-convoluting the mechanisms through which D, Q, or other senolytics exert their effects. Importantly, as we found that senescent cells originating from different types of cells vary in susceptibility to RNA interference and pharmacological interventions, it may be feasible to design drug strategies focused on specific indications by clearing senescent cells arising from particular cell types or in specific organs.

Whether candidate senolytics actually alleviate phenotypes though removing senescent cells or through possible off-target effects on nonsenescent cells is an important and difficult issue to resolve. We first considered comparing effects of senolytics to those of removing senescent cells from *INK-ATTAC* mice. However, we felt that while this may indicate an association between phenotypic effects of removing senescent cells by candidate senolytic drugs and those of removal by activating a ‘suicide gene’ in senescent cells, this approach would not establish cause and effect. Even if candidate senolytic agents had effects resembling those due to genetic clearance of senescent cells, and even if effects of the drugs were not additive to effects of genetic clearance, off-target effects could still not be ruled out. For example, clearing senescent cells genetically could influence a critical effector protein also directly targeted by the drug, especially if studies involve continuous administration of drugs.

We also considered ruling out off-target effects by expressing constitutively active targets of the candidate senolytic drugs in senescent cells of genetically modified mice and determining whether effects of the drugs are blocked in these animals. However, the targets of the senolytic agents we found have important functions in cell regulation, and constitutively expressing them would be anticipated to have many effects that could confound the experiment.

Instead, to start to rule out off-target effects, we examined whether removing senescent cells has sustained effects for many weeks after the drug is no longer present. Apart from agents that permanently alter cellular or tissue composition, such as antimicrobials, anticancer agents, extracellular matrix modifiers, or teratogens, there are few drugs known to exert a sustained effect long after the drugs are no longer present. Indeed, our results demonstrated that a single treatment of D+Q had phenotypic effects persisting far after the drug is no longer present. For example, the treadmill endurance in mice in which one leg had been irradiated 3 months before a single dose of senolytics remained improved to the level of that in sham-irradiated controls for 7 months after treatment with vehicle or D+Q. In addition, the senolytic treatment did not affect endurance of the sham-irradiated controls. This long-lasting effect is more consistent with a change in cellular or tissue composition; in this case, a decrease in senescent cell burden, than an off-target effect on a metabolite, pathway, or physiological parameter that requires continued dosing with a drug.

An important observation is that senolytics appear to alleviate multiple types of dysfunction. The senolytic agents used here enhanced cardiac and vascular function in aging mice, reduced dysfunction caused by localized irradiation, and alleviated skeletal and neurological phenotypes in progeroid mice. Remarkably, in some cases, these drugs did so with only a single course of treatment. In previous work, we and our collaborators found that genetic clearance of senescent cells slowed development of lordokyphosis, cataracts, and lipodystrophy in progeroid mice (Baker *et al*., 2011). Thus, the accumulation of senescent cells in association with a number of diseases, disabilities, and chronological aging likely contribute to the causation and pathophysiology of these problems or their symptoms. Together with chronic, ‘sterile’ inflammation, macromolecular dysfunction, and stem and progenitor cell dysfunction, cellular senescence may contribute to both aging phenotypes and increased susceptibility to a range of chronic diseases.

An advantage of alleviation of symptoms by a single or few doses of senolytics is that they might be given during periods of generally good health, for example, before elective surgery or other circumstances where senescent cell generation could be beneficial. This may help reduce side effects yet still allow senescent cells to be generated when needed, for example, during wound healing (Demaria *et al*., 2014). This possibility merits further study in animal models. Additionally, as senescent cells do not divide, drug resistance would be expected to be less likely than is the case with antibiotics or cancer treatment, in which cells proliferate and so can acquire resistance (Tchkonia *et al*., 2013; Kirkland & Tchkonia, 2014).

We view this work as a first step toward developing senolytic treatments that can be administered safely in the clinic. Several issues remain to be addressed, including some that must be examined well before the agents described here or any other senolytic agents are considered for use in humans. For example, we found differences in responses to RNA interference and senolytic agents among cell types. Effects of age, type of disability or disease, whether senescent cells are continually generated (*e.g*., in diabetes or high-fat diet vs. effects of a single dose of radiation), extent of DNA damage responses that accompany senescence, sex, drug metabolism, immune function, and other interindividual differences on responses to senolytic agents need to be studied.

Detailed testing is needed of many other potential targets and senolytic agents and their combinations. Other dependence receptor networks, which promote apoptosis unless they are constrained from doing so by the presence of ligands, might be particularly informative to study, especially to develop cell type-, tissue-, and disease-specific senolytic agents. These receptors include the insulin, IGF-1, androgen, and nerve growth factor receptors, among others (Delloye-Bourgeois *et al*., 2009; Goldschneider & Mehlen, 2010). It is possible that more existing drugs that act against the targets identified by our RNA interference experiments may be senolytic. In addition to ephrins, other dependence receptor ligands, PI3K, AKT, and serpines, we anticipate that drugs that target p21, probably p53 and MDM2 (because they regulate p21 and serpines), BCL-xL, and related genes will also have senolytic effects. This is especially so as existing drugs that act through these targets cause apoptosis in cancer cells and are in use or in trials for treating cancers, including dasatinib, quercetin, and tiplaxtinin (Gomes-Giacoia *et al*., 2013; Truffaux *et al*., 2014; Lee *et al*., 2015).

Effects of senolytic drugs on healthspan remain to be tested in chronologically aged mice, as do effects on lifespan. Senolytic regimens need to be tested in nonhuman primates. Effects of senolytics should be examined in animal models of other conditions or diseases to which cellular senescence may contribute to pathogenesis, including diabetes, neurodegenerative disorders, osteoarthritis, chronic pulmonary disease, renal diseases, and others (Tchkonia *et al*., 2013; Kirkland & Tchkonia, 2014).

Like all drugs, D and Q have side effects, including hematologic dysfunction, fluid retention, skin rash, and QT prolongation (Breccia *et al*., 2014). An advantage of using a single dose or periodic short treatments is that many of these side effects would likely be less common than during continuous administration for long periods, but this needs to be empirically determined. Side effects of D differ from Q, implying that (i) their side effects are not solely due to senolytic activity and (ii) side effects of any new senolytics may also differ and be better than D or Q. There are a number of theoretical side effects of eliminating senescent cells, including impaired wound healing or fibrosis during liver regeneration (Krizhanovsky *et al*., 2008; Demaria *et al*., 2014). Another potential issue is cell lysis syndrome if there is sudden killing of large numbers of senescent cells. Under most conditions, this would seem to be unlikely, as only a small percentage of cells are senescent (Herbig *et al*., 2006). Nevertheless, this possibility needs to be tested.

Senescent cells have been identified at sites of pathology in multiple diseases and disabilities or may have systemic effects that predispose to others (Tchkonia *et al*., 2013; Kirkland & Tchkonia, 2014). Our findings here give support for the speculation that these agents may one day be used for treating cardiovascular disease, frailty, loss of resilience, including delayed recovery or dysfunction after chemotherapy or radiation, neurodegenerative disorders, osteoporosis, osteoarthritis, other bone and joint disorders, and adverse phenotypes related to chronologic aging. Theoretically, other conditions such as diabetes and metabolic disorders, visual impairment, chronic lung disease, liver disease, renal and genitourinary dysfunction, skin disorders, and cancers could be alleviated with senolytics. (Kirkland, 2013a; Kirkland & Tchkonia, 2014; Tabibian *et al*., 2014). If senolytic agents can indeed be brought into clinical application, they would be transformative. With intermittent short treatments, it may become feasible to delay, prevent, alleviate, or even reverse multiple chronic diseases and disabilities as a group, instead of one at a time.

## Experimental procedures

### Preadipocyte isolation and culture

Detailed descriptions of our preadipocyte, HUVEC, MEF, and MSC culture methods are in Data S1 and publications (Tchkonia *et al*., 2007; Wang *et al*., 2012). The protocol was approved by the Mayo Clinic Foundation Institutional Review Board for Human Research.

### Induction of cellular senescence

Preadipocytes or HUVECs were irradiated with 10 Gy of ionizing radiation to induce senescence or were sham-irradiated. Preadipocytes were senescent by 20 days after radiation and HUVECs after 14 days, exhibiting increased SA-βGal activity and SASP expression by ELISA (IL-6, MCP-1). Where indicated, senescence was induced by serially subculturing cells.

### Microarray analysis

Microarray analyses were performed using the r environment for statistical computing (http://www.R-project.org). Array data are deposited in the GEO database, accession number GSE66236. Gene Set Enrichment Analysis (version 2.0.13) (Subramanian *et al*., 2005) was used to identify biological terms, pathways, and processes that were coordinately up- or down-regulated with senescence. The Entrez Gene identifiers of genes interrogated by the array were ranked according to the *t* statistic. The ranked list was then used to perform a pre-ranked GSEA analysis using the Entrez Gene versions of gene sets obtained from the Molecular Signatures Database (Subramanian *et al*., 2007). Leading edges of pro- and anti-apoptotic genes from the GSEA were performed using a list of genes ranked by the Student *t* statistic.

### Senescence-associated β-galactosidase activity

Cellular SA-βGal activity was quantitated using 8–10 images taken of random fields from each sample by fluorescence microscopy.

### RNA methods

Primers are described in Table S2. Cells were transduced with siRNA using RNAiMAX and harvested 48 h after transduction. RT–PCR methods are in our publications (Cartwright *et al*., 2010). TATA-binding protein (TBP) mRNA was used as internal control.

### Network analysis

Data on protein–protein interactions (PPIs) were downloaded from version 9.1 of the STRING database (PubMed ID 23203871) and limited to those with a declared ‘mode’ of interaction, which consisted of 80% physical interactions, such as activation (18%), reaction (13%), catalysis (10%), or binding (39%), and 20% functional interactions, such as post-translational modification (4%) and co-expression (16%). The data were then imported into Cytoscape (PMID 21149340) for visualization. Proteins with only one interaction were excluded to lessen visual clutter.

### Mouse studies

Mice were male C57Bl/6 from Jackson Labs unless indicated otherwise. Aging mice were from the National Institute on Aging. *Ercc*^1*−/Δ*^ mice were bred at Scripps (Ahmad *et al*., 2008). All studies were approved by the Institutional Animal Care and Use Committees at Mayo Clinic or Scripps.

### Single leg radiation

Four-month-old male C57Bl/6 mice were anesthetized and one leg irradiated with 10 Gy. The rest of the body was shielded. Sham-irradiated mice were anesthetized and placed in the chamber, but the cesium source was not introduced. By 12 weeks, p16 expression is substantially increased under these conditions (Le *et al*., 2010).

### Vasomotor function

Rings from carotid arteries were used for vasomotor function studies (Roos *et al*., 2013). Excess adventitial tissue and perivascular fat were removed, and sections of 3 mm in length were mounted on stainless steel hooks. The vessels were maintained in an organ bath chamber. Responses to acetylcholine (endothelium-dependent relaxation), nitroprusside (endothelium-independent relaxation), and U46619 (constriction) were measured.

### Echocardiography

High-resolution ultrasound imaging was used to evaluate cardiac function. Short- and long-axis views of the left ventricle were obtained to evaluate ventricular dimensions, systolic function, and mass (Roos *et al*., 2013).

### Treadmill endurance

As a measure of physical function, exercise capacity was determined on a motorized treadmill (LeBrasseur *et al*., 2009). Running time was recorded, and running distance (a function of time and speed on the treadmill) and work were calculated.

## Supporting Information

**Fig. S1**The extent of mRNA knock-down by the various siRNAs (A-B) and siRNA transfection (C1–10) were equivalent in senescent preadipocytes (C5,6), proliferating non-senescent preadipocytes (C1,2), differentiated non-senescent preadipocytes (C3,4), proliferating HUVECs (C7,8), and senescent HUVECs (C9,10). Cells were transfected with siRNAs or a siGLO™ Red Transfection Indicator (**C1,3,5,7,9**). **C2,4,6,8,10** show phase contrast images to illustrate cell density. After 48 hours, cells were harvested and mRNA was quantified by RT-PCR. Histograms represent the mean ± SEM for 3 independent replicates. **Fig. S2** Validation of gene expression data (A-B) Confirmation of the siRNA data using a second siRNA to knock-down expression against each of the indicated genes in radiation-induced senescent vs. non-senescent human subcutaneous preadipocytes and HUVECs. (C) Reducing expression of EFNB2 or a PI3K isoform, PI3KCG, distinct from PI3KCD, had no effect on the viability of either senescent or non-senescent preadipocytes. (D) Senescent cells can be selectively targeted by suppressing expression of pro-survival mechanisms using RNA interference in serially-passaged human preadipocytes. Targeting survival pathways by siRNA reduces viability (ATPLite) of serial passage-induced senescent human abdominal subcutaneous primary preadipocytes to a greater extent than non-senescent proliferating cells. **P*<0.05, *N* = 4, *T* test. Primary preadipocytes were serially passaged for 28 population doublings. Senescence was confirmed by SA-βGal assay (75–85%). (E) Senolytic siRNAs did not interfere with the viability of quiescent, differentiated preadipocytes. Confluent human abdominal subcutaneous 4^th^ passage primary preadipocytes were treated with differentiation-inducing medium for 20 days and then cells were transfected with each siRNA using RNAiMAX reagent and maintained in the maintenance media for 2 days. Media were changed back to regular culture media for 5 days. Cell viability was assayed by ATPLite on the 5^th^ day. (F) The expression of EFNB1, EFNB3, PI3KCD, and p21 proteins was reduced to a similar extent by siRNA in senescent and non-senescent preadipocytes. Human abdominal subcutaneous 4^th^ passage primary preadipocytes were transfected with EFNB1, EFNB3, PI3KCD, p21, or scrambled siRNAs. After 48 hours, cells were harvested and protein was assayed by Western immunoanalysis. β-actin was assayed as a loading control. Note that although many anti-apoptotic regulatory pathways were up-regulated in senescent cells by array pathway analysis, not every individual anti-apoptotic regulator was increased. We speculated that the ones that are less highly expressed, including the ephrins and PI3KCD, may be good senolytic targets because by down-regulating them to the same extent in senescent and non-senescent cells we could selectively elicit a functional change (apoptosis) in senescent cells without affecting non-senescent cells. If instead we targeted the most highly expressed anti-apoptotic factors, this would require a greater extent of knock-down to induce apoptosis and is more apt to elicit a non-specific effect in all cells. **Fig. S3** (A) PAI-1 siRNA decreases ATPLite in senescent preadipocytes. This could not be confirmed using a 2nd siRNA. No effect was observed in HUVECs. **P*<0.05, *N* = 6, *T* test. (B) The PAI-1 inhibitor tiplaxtinin exhibited possible senolytic effects at a high concentration, 200 mM, in preadipocytes assayed by ATPLite. N=2 subjects; means±SEM are shown. PAI-1 antagonists are in clinical trials for inducing apoptosis in gastrointestinal and xenografted lung and skin cancers (Mutoh *et al*. 2008; Masuda *et al*. 2013). **Fig. S4** Dasatinib is more senolytic than imatinib. Human subcutaneous preadipocytes were exposed to 10 Gy radiation 25 days before or were non-irradiated. Cells were plated at sub-confluent (50%) density at day 0 (=100%). After 3 days of exposure to dasatinib (**A**) or imatinib (**B**), ATPLite was used to assay viable cells. Note that by day 3, the proliferating cells had increased to ∼190% of plated cells at day 0, while senescent cells did not divide, remaining near 100%. The distance between the red lines (denoting senescent cells plated) and the data points corresponds to % senescent cells eliminated. Dasatinib caused a dose-dependent decrease in senescent cells to ∼50- of those plated, up to the concentration at which it also inhibited non-senescent cell viability (1000 nM), whereas imatinib did not reduce senescent cell viability more than that of proliferating cells at any concentration. Note that imatinib EC50 against cancer cells is ∼10 fold higher than dasatinib (O’Hare et al. 2005). N=4 independent experiments, means±SEM are shown. **Fig. S5** (A) Over 95 percent of radiated cultured human preadipocytes are SA-βGal^+^. A1: Proliferating 4^th^ passage primary human abdominal subcutaneous preadipocytes assayed for SA-βGal (male, 57 years old, BMI 29.5). A2: Fourth passage primary human abdominal subcutaneous preadipocytes 25 days following 10 Gy x-ray radiation when they were assayed for SA-βGal. Representative of 3 experiments. A3: Fifth passage proliferating HUVECs assayed for SA-βGal. A4: Fifth passage HUVECs 10 days following 10 Gy x-ray radiation when they were assayed for SA-βGal. (B) % SA-βGal^+^ cells in cultured non-senescent proliferating vs. radiated vs. serial passage-induced senescent preadipocytes. (C) % SA-βGal^+^ cells in cultured non-senescent proliferating vs. radiation-induced senescent HUVECs. **Fig. S6** Elimination of senescent vs. non-senescent human preadipocytes and HUVECs by siRNA knockdown. **A**: Abdominal subcutaneous primary human preadipocytes were transfected with EFNB-1, EFNB-3, PI3KCD, p21, PAI-2, or scrambled siRNAs. The siRNAs caused death selectively of senescent *vs*. non-senescent preadipocytes. Representative of 5 experiments. Figures **A1-6** are senescent preadipocytes; **A7-12** are non-senescent, proliferating 4^th^ passage preadipocytes. **B**: Radiation-induced senescent HUVECs **(B1-3)** and non-senescent, proliferating HUVECs **(B4-6)** were transfected with EFNB-1, BCL-xL, or scrambled siRNAs. After 5 days, cultures were stained with crystal violet. **Fig. S7** D and Q cause apoptosis in senescent primary human preadipocytes. **A**: DNA fragmented substantially in the nucleus of senescent cells after a 24-48 hour exposure to D+Q (DNA fragmentation circled in red). **B**: Terminal deoxynucleotidyl transferase dUTP nick end labeling (TUNEL) with florescent green dye shows apoptotic cells. **Fig. S8** Leading edges of gene sets at higher resolution. Heat maps of the leading edges of gene sets related to anti-apoptotic function, “negative regulation of apoptosis” **(A)** and “anti-apoptosis” **(B)**, in senescent vs. non-senescent preadipocytes are shown (red = higher; blue = lower). Samples are ordered from left to right first by proliferative state and then by subject (N=8), and the rows are ordered from top to bottom by the absolute value of the Student t statistic computed between the senescent and proliferating cells (*i.e*., from greatest to least significance). Each column represents one subject. **Fig. S9** Periodic or acute treatment of *Ercc1*^*−/Δ*^ mice with D+Q attenuates senescence in tissues of *Ercc1*^*−/Δ*^ mice. **A**: Liver sections from 4 mice, 2 treated weekly with D+Q and 2 that received vehicle only (V) stained for SA-βGal (5X magnification). **B**: Quantitation of the number of SA-βGal+ cells in ten random fields of each section, normalized to the vehicle only group. *p=0.032, Student’s t test. C: SA-βGal staining of fat from 4 mice, 2 treated acutely with D+Q (3 doses in one week M,W,F) and 2 that received vehicle only. Interestingly, more senescence was detected in male *Ercc1*^-/D^ mice, affording greater differences in male mice treated with D+Q than female mice (Fig. S12). **Fig. S10** Representative images of *Ercc1*^*−/Δ*^ mice from the D+Q treatment group or vehicle only. Splayed feet are an indication of dystonia and ataxia. Images of 15 week old *Ercc1*^-/δ^p mice **(A-D)**. Aligned left and right are age-matched animals treated periodically with vehicle only or D+Q. Signs of aging and frailty that appear improved in the D+Q group are indicated with arrows. Images of 16 week old *Ercc1*^-/D^ mice **(E-H)**. Aligned left and right are sibling pairs treated periodically with vehicle only or D+Q. Signs of aging and frailty that appear improved in the D+Q group are indicated with arrows. **Fig. S11** Pairwise analysis of the age at onset of all age-related symptoms measured in sibling pairs of *Ercc1*^*−/Δ*^ mice. Each color represents a different symptom. The height of the bar indicates the severity of the symptom at a particular age. The composite height of the bar is an indication of the animals’ overall health (lower=better health). Many symptoms are delayed in onset in the *Ercc1*^-/D^ mice treated with D+Q compared to siblings treated with vehicle only, for example ataxia (orange), grip strength (red), hind limb paralysis (green), and gait disorder (dark blue). Many symptoms are also attenuated, for example dystonia (light blue), gait disorders (dark blue), and ataxia (orange). **Fig. S12** Differences in the response of male and female *Ercc1*^*−/Δ*^ mice to D+Q. **A**: Male *Ercc1*^-/D^ mice have higher expression of the senescence marker p16 mRNA in kidney lysates. Therefore the impact of D+Q on p16 expression is more dramatic in male mice. **B**: Glycosaminoglycan expression is significantly increased in the intervertebral disc nucleus pulposa of male *Ercc1*^-/D^ mice. Sexual dimorphisms need to be investigated in the future with larger cohorts of mice. *p<0.03, Student?s t test. **Fig. S13** Representative images of the tissue sections scored for age-related histopathological changes. Both sections are from a female mouse treated weekly with 5 mg/kg Dasatinib and 50 mg/kg Quercetin for 10 weeks then euthanized at 16 weeks of age. Shown are hematoxylin and eosin stained sections of the liver and kidney imaged at 200X. Hepatopathy: the liver shows a combination of degenerative changes, including sporadic necrosis, regenerative changes, and markedly distended hepatocytes with glycogen and with or without discrete membrane-bound lipid inclusions. Nephropathy: the kidney shows a combination of minor sclerotic changes in a glomerulus, mild inflammation with cell infiltration, and anisonucleosis in the renal tubular epithelium. **Table S1** siRNAs screened for senolytic activity. 39 siRNAs were screened by ATPLite assay for effects on senescent and non-senescent preadipocyte and HUVEC viability. **Table S2** Human and mouse TagMan primer probes used. **Table S3** Effects of senolytic treatment (D+Q) on cardiac function. **Data S1** Materials and methods.
