# Supplementary material for: The Achilles’ heel of senescent cells: from transcriptome to senolytic drugs
Source: Aging Cell. 2015 Apr 22;14(4):644–58. doi: 10.1111/acel.12344 (PMC4531078; doi:10.1111/acel.12344)
Supplement: Supplementary file 1 [file acel0014-0644-sd1.zip › Supporting Information Methods.docx]

**Data Supplement 1. Materials and Methods**

*Preadipocyte isolation and culture*

Fat tissue for preadipocyte isolation was obtained during intra-abdominal surgery from 43 subjects; 6 male, 37 female; age 41.3±1.90 (means±SEM) years, who had given informed consent. The protocol was approved by the Mayo Clinic Foundation Institutional Review Board for Human Research. Detailed descriptions of preadipocyte and adipose-derived endothelial cell culture systems are in our publications ([Caserta *et al.* 2001](#_ENREF_5); [Karagiannides *et al.* 2001](#_ENREF_14); [Tchkonia *et al.* 2002](#_ENREF_27); [Tchkonia *et al.* 2005](#_ENREF_30); [Karagiannides *et al.* 2006](#_ENREF_15); [Tchkonia *et al.* 2006](#_ENREF_28); [Tchkonia *et al.* 2007](#_ENREF_29)). Primary culture studies can be complicated by the presence of other cell types, heterogeneous subpopulations, and variable recovery. We addressed these issues. We optimized digestion and plating and demonstrated our methods yield essentially pure preadipocyte populations, irrespective of age or depot origin ([Wang *et al.* 1989](#_ENREF_34); [Kirkland *et al.* 1990](#_ENREF_16); [Kirkland *et al.* 1993](#_ENREF_17); [Kirkland *et al.* 1994](#_ENREF_18); [Tchkonia *et al.* 2002](#_ENREF_27)). We used differential plating to prevent macrophage or endothelial cell contamination (both are more trypsin-resistant than preadipocytes; macrophages, mesenchymal progenitors, and endothelial cells do not proliferate well in media optimized for preadipocytes). Following differential plating, macrophage and endothelial cell markers did not differ consistently with age or among depots. Under our conditions for isolating and culturing cells, mesenchymal stem cell markers are not detectible (Nanog, Oct3/4, Cd34, Cd117, Sox2, Smad4, Abcg2, Utf1, Sca1). Discussion about cell type markers in fat tissue and controversies about nomenclature were reviewed by us recently ([Tchkonia *et al.* 2013](#_ENREF_31)). All new primary cultures were tested for Mycoplasma.

*Human Umbilical Vein Endothelial cell (HUVEC) culture and reagents*

Human umbilical vein endothelial cells (HUVECs) were purchased from Lonza and grown in Clonetics Endothelial Cell Growth Medium-2 (EGM-2; Lonza, Walkersville, MD) according to the protocol provided by the company ([Wang *et al.* 2012](#_ENREF_35)).

*Induction of cellular senescence*

Preadipocytes or HUVEC’s were radiated in RS2000 X-Ray Irradiator (RAD Source Technologies) at 10 Gy to induce senescence or were sham-radiated. Preadipocytes were senescent by 20 days after radiation and HUVEC’s after 14 days, exhibiting SA-βGal positivity and SASP factor expression by ELISA (IL-6, MCP-1). Alternatively, senescence was induced by serially subculturing cells. For preadipocytes, cells were passaged at a 1:2 split ratio until growth slowed. Senescence was confirmed by SA-βGal and BrDU staining. Both non-senescent and senescent preadipocytes and HUVECs were plated at 10^4^ cells/cm^2^ in each experiment.

*Chemicals*

Dasatinib was purchased from LC Laboratories (cat# D-3307, Woburn, MA). Quercetin was purchased from Sigma (cat# 1592409, St. Louis, MO). Tiplaxtinin was purchased from Axon Medchem (cat# Axon 1383, Reston, VA).

*MSC isolation*

MSC were obtained from *Ercc1*^-/Δ^ mouse bone marrow and cultured in high glucose DMEM supplemented with 15% FBS, 2mM glutamine, 100 U/ml penicillin, and 0.1 mg/ml streptomycin (all from Sigma-Aldrich, St Louis, MO, USA). The MSC were cultured in low oxygen conditions (3% O_2_) to avoid oxidative damage. The generated MSC displayed a CD105 +, CD106 +, CD73 +, Sca-1+, CD34-, CD45-, and CD31- phenotype, fibroblast-like morphology, and multi-lineage differentiation capacity. Senescence was induced by continuous passaging and the cells used for drug testing at passage 5.

*MEF isolation*

The *Ercc1*^-/-^ MEFs were isolated from pregnant females at ~day 13 post-coitus and cultured in a 1:1 mixture of Dulbecco’s modified Eagle’s medium and Ham’s F10 with 10% fetal bovine serum, 1x nonessential amino acids, penicillin, and streptomycin and incubated at 3% 0_2_ initially, followed by a shift to 20% for 5 passages to induce senescence.

*Analysis of the effects of drugs on senescence in MEFs*

5x10^3^ cells from MEFs at passage 5 at 20% O_2_ were seeded per well in a 96 well plate 6 hours prior to treatment. Following addition of the drugs, the MEFs were incubated for 48 hours under 20% O_2_ oxygen conditions. For the fluorescence analysis of SA-ßGal activity, the cells were washed 1x with PBS, C_12_FDG (10 µM) added to the culture medium, and the cells incubated for 1.5 – 2 hours. Ten minutes prior to analysis, the DNA intercalating Hoechst dye (2 µg/ml) was added to the cells. An In Cell Analyzer 6000, a laser-based line scanning confocal imager with a large field-of view sCMOS camera detection technology, was used for the quantitative analysis of cell number (Hoechst staining) and the number of C_12_FDG positive senescent cells.

*Cell viability, cell death, and apoptosis assays in preadipocytes and HUVECs*

Cell viability after treatment was measured by ATPLite Kit (cat# 6016941, PerkinElmer, Waltham, MA). The assay was performed following the manufacturer’s instructions. Luminescence was read using a multi scan plate reader (Fisher, Waltham, MA). Cell death was measured with crystal violet. Cells were washed twice with PBS and then incubated with PBS containing 1% paraformaldehyde for 15 minutes at room temperature and stained with 0.1% crystal violet for 15 minutes at room temperature. Cells were washed with deionized water and staining intensity was measured with a spectrometer at λ540. Apoptosis assay was performed using DeadEnd™ Fluorometric TUNEL assay (Promega, Madison, WI). Cells were cultured on 8-well tissue culture chamber slides (BD Falcon, Bedford, MA) and then treated with either DMSO or drugs for 8-24 hours. The assays were performed following the manufacturer’s instructions.

*Drug screening assay*

Proliferating non-senescent human primary preadipocytes or HUVECs and senescent cells induced by 10 Gy radiation were plated at same cell densities in different wells in 96-well plates. Senescence was confirmed by SA-βGal staining and the percentage of SA-βGal+ cells was over 80% (Supplemental Fig. 5). Cell viability at day 0 was measured by ATPLite assay and drugs or vehicle were then added into each well. After 3 days exposure, the cell viability was measured by ATPLite.

*Microarray analysis*

Raw Affymetrix CEL files were normalized to produce gene-level expression values using the implementation of the Robust Multiarray Average (RMA)([Irizarry *et al.* 2003](#_ENREF_13)) in the affy package (version 1.36.1)([Gautier *et al.* 2004](#_ENREF_8)) included within in the Bioconductor software suite (version 2.12)([Gentleman *et al.* 2004](#_ENREF_9)) and an Entrez Gene-specific probeset mapping (version 16.0.0) from the Molecular and Behavioral Neuroscience Institute (Brainarray) at the University of Michigan([Dai *et al.* 2005](#_ENREF_6))( http://brainarray.mbni.med.umich.edu/Brainarray/Database/CustomCDF). Array quality was assessed by computing Relative Log Expression (RLE) and Normalized Unscaled Standard Error (NUSE) using the affyPLM Bioconductor package (version 1.34.0) (Brettschneider J, Collin F, Bolstad BM, and Speed TP. Quality Assessment for Short Oligonucleotide Microarray Data. Technometrics 2008; 50(3):241. DOI 10.1198/004017008000000334). Principal Component Analysis (PCA) was performed using the 'prcomp' R function with expression values that had been normalized across all samples to a mean of zero and a standard deviation of one. Differential gene expression was assessed by performing t tests on the coefficients of simple linear models (equivalent to a two-way ANOVA) created using the limma package (version 3.14.4) (Smyth, G. K. (2005). Limma: linear models for microarray data. In: 'Bioinformatics and Computational Biology Solutions using R and Bioconductor'. R. Gentleman, V. Carey, S. Dudoit, R. Irizarry, W. Huber (eds), Springer, New York, pages 397-420). Correction for multiple hypothesis testing was accomplished using the Benjamini-Hochberg false discovery rate (FDR)([Benjamini & Hochberg 1995](#_ENREF_3)). All microarray analyses were performed using the R environment for statistical computing (version 2.15.1) (http://www.R-project.org)(R Development Core Team (2011). R: A language and environment for statistical computing. R Foundation for Statistical Computing, Vienna, Austria. ISBN 3-900051-07-0). All array data are deposited in the GEO database: accession number GSE66236.

*Gene Set Enrichment Analysis (GSEA)*

GSEA (version 2.0.13)([Subramanian *et al.* 2005](#_ENREF_24)) was used to identify biological terms, pathways, and processes that were coordinately up- or down-regulated with senescence. The Entrez Gene identifiers of the genes interrogated by the array were ranked according to the t statistic. The ranked list was then used to perform a pre-ranked GSEA analysis (default parameters with random seed 1234) using the Entrez Gene versions of the Biocarta, KEGG, Reactome, Gene Ontology (GO), and transcription factor and microRNA motif gene sets obtained from the Molecular Signatures Database (MSigDB), version 4.0([Subramanian *et al.* 2007](#_ENREF_23)). Leading edges of pro- and anti-apoptotic genes from the GSEA were performed using a list of genes ranked by the Student t statistic.

*Senescence-associated β-galactosidase activity*

Cellular SA-βGal activity was assayed as previously described ([Stout *et al.* 2014](#_ENREF_22)). In brief, primary preadipocytes or fat tissues were washed with PBS 3 times and then fixed in PBS containing 2% formaldehyde (Sigma-Aldrich, St. Louis, MO, USA) and 0.25% glutaraldehyde (Sigma-Aldrich) for 10 minutes. Following fixation, cells or tissues were washed with PBS 3 times before being incubated in SA-βGal activity solution (pH 6.0) at 37^o^C for 16-18 hours. The enzymatic reaction was stopped by washing cells or tissues with ice-cold PBS 3-5 times. Images were made by fluorescence microscopy (Nikon Eclipse Ti, Melville, NY). From 8-10 images were taken of random fields from each sample.

*Small interfering (si) RNA knockdown*

siRNA oligonucleotide duplexes targeting EFNB1, EFNB3, PI3K CD, p21(CDKN1A), PAI-1, PAI-2, and BCL-xL were purchased from Life Technologies. Cells were transfected using RNAiMAX reagent following the manufacture’s protocol (Life Technologies, Grand Island, NY). Transfection efficiencies were monitored by a siGLO™ Red Transfection Indicator (GE Healthcare Dharmacon, Lafayette, CO). The IDs of each siRNA are as follows: EFNB1, 1st: ID #14628; 2nd: ID# HSS103091; EFNB3, 1^st^: ID#145856; 2nd: ID# HSS103096; PI3K CD, 1st: ID#15717; 2nd: ID#143976; P21, 1^st^: ID#1436; 2nd: ID#118565; PAI-1, 1st: ID#s10013; 2nd: ID#s10015; PAI-2, 1st: ID# 118663; 2nd: ID#4240; BCL-xL, 1st: ID#120716; 2nd: ID#120717; PIK3CG, ID#11879; EFNB2, ID#14087.

*Western blot analysis and immunodetection of senescence markers*

25µg total protein isolated from cell cultures were loaded per lane for Western blot analyses. Antibodies used in the study were rabbit anti-EFNB1 (1:1000, NBP1-60608, Novus Biologicals, Littleton, CO), mouse anti-EFNB3 (1 µg/mL, MAB395, R&D system, Minneapolis, MN), mouse anti- PI3Kinase p110δ (1 µg/mL, MAB2687, R&D system, Minneapolis, MN), rabbit anti-p21 (1:500, sc-397, Santa Cruz Biotechnology, Dallas, Texas), rabbit anti-Bcl-xL (1:1000, #2764, Cell Signaling, Danvers, MA), mouse anti-SerpinB2 (PAI-2) (1:200, NBP2-01492, Novus, Littleton, CO), and rabbit anti-GADPH (1:1000,#5174, Cell Signaling, Danvers, MA). Flash frozen tissue was weighed and homogenized in RIPA buffer (Thermo Scientific, Pittsburgh, PA) containing the Complete mini-protease inhibitor cocktail and PhosSTOP (phosphatase inhibitor) (Roche Molecular Biochemicals, Branchburg, NJ). From each sample, 30 µg of protein was resolved on 4-20% Mini Protean TGX polyacrylamide gels (Bio-Rad). p21 was detected with rabbit anti-p21 (1:500, Abcam, Cambridge, MA). β-actin (1:3000, Cell signaling, Beverly, MA) was used as a loading control. Secondary antibody was anti-rabbit HRP (1:5000, Invitrogen, Carlsbad, CA) or rabbit anti-β-actin (1:1000, 8457, Cell Signaling Technology). The secondary antibodies included anti-rabbit or anti-mouse IgG obtained from Santa Cruz, CA.

*Real-time PCR*

RT- PCR methods are in our publications ([Cartwright *et al.* 2010](#_ENREF_4)). Briefly, RNA from cells was extracted using RNeasy Plus Mini Kit (Qiagen) and was reverse transcribed to cDNA using M-MLV Reverse Transcriptase kit (Invitrogen). Probes and TaqMan fast advanced master mix were purchased from Applied Biosystems. TATA-binding protein (TBP) was used as internal control. Real-time PCR was performed 7500 Fast Real-Time PCR System (Applied Biosystems). Primers are in Supplemental Table 2.

*Mouse studies*

Mice were male C57Bl/6 from Jackson Labs unless indicated otherwise. Aging mice were from the National Institute on Aging. *Ercc^-/Δ^* mice were bred at Scripps. All studies were approved by the Institutional Animal Care and Use Committees at Mayo Clinic or Scripps. Ercc1^-/∆^ mice express ~5% of the normal complement of the DNA repair endonuclease ERCC1-XPF. As a consequence, the mice accumulate endogenous oxidative DNA damage more rapidly than normal mice and age 6X faster than normal mice ([Dolle *et al.* 2011](#_ENREF_7)). The animals also spontaneously develop numerous age-related chronic degenerative diseases ([Gregg *et al.* 2011](#_ENREF_10)). *Ercc1*^-/∆^ mice are bred in a f1 background (C57Bl/6:FVB/n) and genotyped as previously described([Ahmad *et al.* 2008](#_ENREF_2)).

*Single leg radiation*

Four month old male C57/Bl6 mice were anesthetized with isofluorane and then one leg radiated with 10 Gy using ^137^cesium. The rest of the body was shielded. Sham radiated mice were also anesthetized and placed in the machine, but the cesium source was not introduced into the chamber. By 12 weeks, p16 expression is substantially increased under these conditions ([Le *et al.* 2010](#_ENREF_19)).

*Vasomotor function*

Rings from carotid arteries were used for vasomotor function studies, as described previously ([Roos *et al.* 2013](#_ENREF_21)). Excess adventitial tissue and perivascular fat were removed, and sections of approximately 3 mm in length were mounted on stainless-steel hooks. The vessels were maintained in an organ bath chamber containing oxygenated (95% O_2_/5% CO_2_) Krebs solution at 37°C. Responses to acetylcholine (endothelium-dependent relaxation), nitroprusside (endothelium-independent relaxation), and U46619 (constriction) were evaluated.

*Echocardiography*

High-resolution ultrasound imaging (Vevo 2100, Visual Sonics) was used to evaluate cardiac function. Short- and long-axis views of the left ventricle were obtained to evaluate ventricular dimensions, systolic function, and mass ([Roos *et al.* 2013](#_ENREF_21)).

*Treadmill endurance*

As a measure of physical function, exercise capacity was determined on a motorized treadmill (Columbus Instruments, Columbus, OH) as previously described ([LeBrasseur *et al.* 2009](#_ENREF_20)). In brief, mice were acclimated to the treadmill for 3 consecutive days for 5 min at a speed of 8 m/min and a grade of 5%. The next day, mice ran at an initial speed of 8 m/min and grade of 5% for 3 min. The speed was then increased by 2 m/min every 3 min until the mice were exhausted. Exhaustion was defined as the inability of the mouse to remain on the treadmill despite an electrical shock stimulus and mechanical prodding. Running time was recorded, and running distance (a function of time and speed of the treadmill) and work (the product of body weight [kg], gravity [9.81 m/s^2^], vertical speed [m/s × angle], and time [s]) were calculated.

*Periodic treatment study*

*Ercc1^-/Δ^* mice, which model a human progeroid syndrome, were treated weekly with a combination of Dasatanib and Quercetin (5 mg/kg and 50 mg/kg, respectively). The therapeutics were administered in 10% PEG400 by oral gavage weekly to *Ercc1^-/Δ^* mice, beginning at 4-6 weeks of age. Litters with multiple *Ercc1^-/Δ^* mice were used to enable comparison of sibling pairs treated with the therapeutic *vs.* vehicle only. Animal weights were measured bi-weekly. There was no difference in the percent change in body weight for the two treatment groups (data not shown). Animals were scored twice per week for the onset of progeroid symptoms including kyphosis due to osteoporosis, tremor, dystonia, coat condition, ataxia, loss of grip strength, body condition, gait disorders, hind limp paralysis, and urinary incontinence, as previously described ([Tilstra *et al.* 2012](#_ENREF_32)). The investigator doing the analysis was blinded as to the treatment group. All symptoms were scored on a scale of 0, 0.5, or 1.0 indicates no indication of symptom. 0.5 indicates the symptom was detected but not consistently expressed. A score of 1 indicates when the animal begins to express the symptom 100% of the time. Dystonia is the only symptom that was scored on a scale of 0-5 to grade its progression. Thus each animal could have a maximal score of 14 indicating full expression of all age-related symptoms. There were 7 animals in the treatment group and 8 in the vehicle only group. For the post-mortem analysis, all mice were included. For the pre-mortem analysis, only mice for which there is a sex-matched control (vehicle only) that started treatment at the same age were used (N=6 D+Q; N=7 vehicle).

*Histopathology*

Hematoxylin and eosin stained sections of the liver, kidney, and femoral bone marrow space were scored for age-related pathology. Diagnosis of each histopathological change and severity of major lesions were determined with histological classifications in aging mice previously described ([Ikeno *et al.* 2003](#_ENREF_11); [Ikeno *et al.* 2005](#_ENREF_12)). Hepatopathy was scored in the liver, referring to a combination of degenerative changes, including sporadic necrosis, and regenerative changes. Nephropathy was scored in the kidney, referring to a combination of minor sclerotic change, mild inflammation with cell infiltration, and anisonucleosis in the renal tubular epithelium. The femoral sections were scored for bone marrow hypoplasia. Each sample was scored on a scale of 0-4 (0=normal, 1=minimal, 2=mild, 3=moderate, 4=severe). All pathological analyses were accomplished by a double blind procedure without knowledge of the animal’s identity. Two pathologists made similar, independent diagnoses in most cases.

*Quantitation of glycosaminoglycans*

Loss of glycosaminoglycan (GAG) content of the nucleous pulposus (NP) of the intervertebral disc (IVD) is a pronounced age-related phenotype in *Ercc1^-/Δ^* mice ([Vo *et al.* 2010](#_ENREF_33)) that is highly responsive to therapeutic intervention ([Tilstra *et al.* 2012](#_ENREF_32)). NP tissue was isolated and pooled from five lumbar IVDs of each mouse. The pooled tissue sample was digested using papain at 60°C for 2h. GAG content was measured in using dimethylmethylene blue using chondroitin-6-sulfate (Sigma, Milwaukee, WI, C-8529) as a standard. The DNA concentration of each sample was measured using the PicoGreen assay (Molecular Probes, Sunnyvale, CA) and used to normalize the GAG values, as previously described ([Vo *et al.* 2010](#_ENREF_33)). Average values from six reaction samples (two duplicates × three mice per group) were calculated with 1 SE.

*Quantitative computed tomography (pQCT)-derived bone density measurements*

pQCT measurements of the lumbar spine were performed using the Stratec XCT Research SA Plus using software version 5.40 (Norland Medical Systems, Fort Atkinson, WI, USA). The CT speed was set at 3 mm/sec, pixel size was 70 µm x 70 µm, and slice thickness was 0.5 mm. *Ex vivo* lumbar spine scans were performed by placing the spines in a plastic tube filled with 70% ethanol with the dorsal surface facing upward. Total and trabecular bone parameters were calculated as previously described ([Syed & Melim 2011](#_ENREF_25)). The threshold for trabecular bone was set at 480 mg/cm3 and the cortical bone threshold was set at 710 mg/cm3. CV was 4.4% for total volumetric bone mineral density (vBMD).

*Liver SA-β Gal staining*

Fresh liver tissues were fixed in 10% neutral buffered formalin (NBF) for 3-4 hours at 4 degree, followed by incubation in 30% sucrose overnight at 4 degrees. Liver tissues were then embedded in OCT and cryo-sectioned at 5μm for further staining. Liver slides were washed with PBS 3 times and then incubated in SA-βgal staining solution (pH 5.8) at 37 degrees for 16-24 hours. Ten images were taken randomly using a bright-field microscopy and the number of SA-βgal+ cells was counted. Quantification is mean ±SD.

*Real-time PCR in kidney*

RNA was extracted from kidney using Trizol. Real-time PCR was performed using power SYBR green RT-PCR mix on a StepOnePlus real-time PCR system. Actb (β-actin) was used for normalization of target gene expression.

*Fluorescent in situ hybridization (FISH)*

Detection of p16 mRNA in biliary epithelia was performed as described previously ([Tabibian *et al.* 2014](#_ENREF_26)). Briefly, liver sections were deparaffinized, rehydrated, and boiled in sodium citrate buffer. Slides were prehybridized in a 4X SSC solution containing 3% BSA at 55°C. Slides were then incubated with either a scrambled non-specific probe or a custom designed p16 LNA probe (5’ TCTCATGCCATTCCTTTCCTGT, Exiqon, Woburn, MA) diluted in hybridization buffer containing 10% dextran sulfate in 4X SSC. Slides were hybridized at 55°C for 1 hour and then submitted to a series of 5 washes of decreasing stringency. No fewer than 5 bile ducts per animal (10 control untreated mice, 8 mice each for D, Q, and D+Q) were imaged using confocal scanning laser microscopy. Adobe Photoshop CS3 (Adobe Systems, San Jose, CA) was used to quantitate fluorescence intensity within biliary epithelia ([Agley *et al.* 2012](#_ENREF_1); [Tabibian *et al.* 2014](#_ENREF_26)). Mean fluorescence intensity with standard deviation is shown. A Mann Whitney U Test was performed for each treatment *vs*. control.

**References**

Agley CC, Velloso CP, Lazarus NR, Harridge SD (2012). An image analysis method for the precise selection and quantitation of fluorescently labeled cellular constituents: application to the measurement of human muscle cells in culture. *The journal of histochemistry and cytochemistry : official journal of the Histochemistry Society*. **60**, 428-438.

Ahmad A, Robinson AR, Duensing A, van Drunen E, Beverloo HB, Weisberg DB, Hasty P, Hoeijmakers JH, Niedernhofer LJ (2008). ERCC1-XPF endonuclease facilitates DNA double-strand break repair. *Mol. Cell. Biol.* **28**, 5082-5092.

Benjamini Y, Hochberg Y (1995). Controlling the false discovery rate: A practical and powerful approach to multiple testing. *J. Royal Stat. Soc.* **57**, 289-300.

Cartwright M, Tchkonia T, Lenburg M, Pirtskhalava T, Cartwright A, Lopez MMA, Frampton G, Kirkland JL (2010). Aging, fat depot origin, and fat cell progenitor expression profiles: Setting the stage for altered fat tissue function. *J. Gerontol.* **65**, 242-251.

Caserta F, Tchkonia T, Civelek V, Prentki M, Brown NF, McGarry JD, Forse RA, Corkey BE, Hamilton JA, Kirkland JL (2001). Fat depot origin affects fatty acid handling in cultured rat and human preadipocytes. *Am. J. Physiol.* **280**, E238-E247.

Dai M, Wang P, Boyd AD, Kostov G, Athey B, Jones EG, Bunney WE, Myers RM, Speed TP, Akil H, Watson SJ, Meng F (2005). Evolving gene/transcript definitions significantly alter the interpretation of GeneChip data. *Nucleic Acids Res.* **33**, e175.

Dolle ME, Kuiper RV, Roodbergen M, Robinson J, de Vlugt S, Wijnhoven SW, Beems RB, de la Fonteyne L, de With P, van der Pluijm I, Niedernhofer LJ, Hasty P, Vijg J, Hoeijmakers JH, van Steeg H (2011). Broad segmental progeroid changes in short-lived Ercc1(-/Delta7) mice. *Pathobiol Aging Age Relat Dis*. **1**.

Gautier L, Cope L, Bolstad BM, Irizarry RA (2004). affy--analysis of Affymetrix GeneChip data at the probe level. *Bioinformatics*. **20**, 307-315.

Gentleman RC, Carey VJ, Bates DM, Bolstad B, Dettling M, Dudoit S, Ellis B, Gautier L, Ge Y, Gentry J, Hornik K, Hothorn T, Huber W, Iacus S, Irizarry R, Leisch F, Li C, Maechler M, Rossini AJ, Sawitzki G, Smith C, Smyth G, Tierney L, Yang JY, Zhang J (2004). Bioconductor: open software development for computational biology and bioinformatics. *Genome Biol.* **5**, R80.

Gregg SQ, Robinson AR, Niedernhofer LJ (2011). Physiological consequences of defects in ERCC1-XPF DNA repair endonuclease. *DNA Repair (Amst)*. **10**, 781-791.

Ikeno Y, Bronson RT, Hubbard GB, Lee S, Bartke A (2003). Delayed occurrence of fatal neoplastic diseases in ames dwarf mice: correlation to extended longevity. *The journals of gerontology. Series A, Biological sciences and medical sciences*. **58**, 291-296.

Ikeno Y, Hubbard GB, Lee S, Richardson A, Strong R, Diaz V, Nelson JF (2005). Housing density does not influence the longevity effect of calorie restriction. *The journals of gerontology. Series A, Biological sciences and medical sciences*. **60**, 1510-1517.

Irizarry RA, Hobbs B, Collin F, Beazer-Barclay YD, Antonellis KJ, Scherf U, Speed TP (2003). Exploration, normalization, and summaries of high density oligonucleotide array probe level data. *Biostatistics*. **4**, 249-264.

Karagiannides I, Tchkonia T, Dobson DE, Steppan CM, Cummins P, Chan G, Salvatori K, Hadzopoulou-Cladaras M, Kirkland JL (2001). Altered expression of C/EBP family members results in decreased adipogenesis with aging. *Am. J. Physiol.* **280**, R1772-R1780.

Karagiannides I, Thomou T, Tchkonia T, Pirtskhalava T, Kypreos KE, Cartwright A, Dalagiorgou G, Lash TL, Farmer SR, Timchenko NA, Kirkland JL (2006). Increased CUG triplet repeat binding protein-1 predisposes to impaired adipogenesis with aging. *J. Biol. Chem.* **281**, 23025-23033.

Kirkland JL, Hollenberg CH, Gillon WS (1990). Age, anatomic site, and the replication and differentiation of adipocyte precursors. *Am. J. Physiol.* **258**, C206-C210.

Kirkland JL, Hollenberg CH, Gillon WS (1993). Ageing, differentiation, and gene expression in rat epididymal preadipocytes. *Biochem. Cell Biol.* **71**, 556-561.

Kirkland JL, Hollenberg CH, Kindler S, Gillon WS (1994). Effects of age and anatomic site on preadipocyte number in rat fat depots. *J. Gerontol.* **49**, B31-B35.

Le ON, Rodier F, Fontaine F, Coppe JP, Campisi J, DeGregori J, Laverdiere C, Kokta V, Haddad E, Beausejour CM (2010). Ionizing radiation-induced long-term expression of senescence markers in mice is independent of p53 and immune status. *Aging Cell*. **9**, 398-409.

LeBrasseur NK, Schelhorn TM, Bernardo BL, Cosgrove PG, Loria PM, Brown TA (2009). Myostatin inhibition enhances the effects of exercise on performance and metabolic outcomes in aged mice. *J Gerontol A Biol Sci Med Sci*. **64**, 940-948.

Roos CM, Hagler M, Zhang B, Oehler EA, Arghami A, Miller JD (2013). Transcriptional and phenotypic changes in aorta and aortic valve with aging and MnSOD deficiency in mice. *American journal of physiology. Heart and circulatory physiology*. **305**, H1428-1439.

Stout MB, Tchkonia T, Pirtskhalava T, Palmer AK, List EO, Berryman DE, Lubbers ER, Escande C, Spong A, Masternak MM, Oberg AL, LeBrasseur NK, Miller RA, Kopchick JJ, Bartke A, Kirkland JL (2014). Growth hormone action predicts age-related white adipose tissue dysfunction and senescent cell burden in mice. *Aging (Milano)*.

Subramanian A, Kuehn H, Gould J, Tamayo P, Mesirov JP (2007). GSEA-P: a desktop application for Gene Set Enrichment Analysis. *Bioinformatics*. **23**, 3251-3253.

Subramanian A, Tamayo P, Mootha VK, Mukherjee S, Ebert BL, Gillette MA, Paulovich A, Pomeroy SL, Golub TR, Lander ES, Mesirov JP (2005). Gene set enrichment analysis: a knowledge-based approach for interpreting genome-wide expression profiles. *Proceedings of the National Academy of Sciences of the United States of America*. **102**, 15545-15550.

Syed FA, Melim T (2011). Rodent models of aging bone: an update. *Curr Osteoporos Rep*. **9**, 219-228.

Tabibian JH, O'Hara SP, Splinter PL, Trussoni CE, LaRusso NF (2014). Cholangiocyte senescence by way of N-ras activation is a characteristic of primary sclerosing cholangitis. *Hepatology*. **59**, 2263-2275.

Tchkonia T, Giorgadze N, Pirtskhalava T, Tchoukalova Y, Karagiannides I, Forse RA, DePonte M, Stevenson M, Guo W, Han J, Waloga G, Lash TL, Jensen MD, Kirkland JL (2002). Fat depot origin affects adipogenesis in primary cultured and cloned human preadipocytes. *Am. J. Physiol.* **282**, R1286-R1296.

Tchkonia T, Giorgadze N, Pirtskhalava T, Thomou T, DePonte M, Koo A, Forse RA, Chinnappan D, Carmen Martin-Ruiz C, von Zglinicki T, Kirkland JL (2006). Fat depot-specific characteristics are retained in strains derived from single human preadipocytes. *Diabetes*. **55**, 2571-2578.

Tchkonia T, Lenburg M, Thomou T, Giorgadze N, Frampton G, Pirtskhalava T, Cartwright A, Cartwright M, Flanagan J, Karagiannides I, Gerry N, Forse RA, Tchoukalova Y, Jensen MD, Pothoulakis C, Kirkland JL (2007). Identification of depot-specific human fat cell progenitors through distinct expression profiles and developmental gene patterns. *Am. J. Physiol.* **292**, E298-E307.

Tchkonia T, Tchoukalova YD, Giorgadze N, Pirtskhalava T, Karagiannides I, Forse RA, Koo A, Stevenson M, Chinnappan D, Cartwright A, Jensen MD, Kirkland JL (2005). Abundance of two human preadipocyte subtypes with distinct capacities for replication, adipogenesis, and apoptosis varies among fat depots. *Am. J. Physiol.* **288**, E267-E277.

Tchkonia T, Thomou T, Zhu Y, Karagiannides I, Pothoulakis C, Jensen MD, Kirkland JL (2013). Mechanisms and metabolic implications of regional differences among fat depots. *Cell Metab*.

Tilstra JS, Robinson AR, Wang J, Gregg SQ, Clauson CL, Reay DP, Nasto LA, St Croix CM, Usas A, Vo N, Huard J, Clemens PR, Stolz DB, Guttridge DC, Watkins SC, Garinis GA, Wang Y, Niedernhofer LJ, Robbins PD (2012). NF-kappaB inhibition delays DNA damage-induced senescence and aging in mice. *The Journal of clinical investigation*. **122**, 2601-2612.

Vo N, Seo HY, Robinson A, Sowa G, Bentley D, Taylor L, Studer R, Usas A, Huard J, Alber S, Watkins SC, Lee J, Coehlo P, Wang D, Loppini M, Robbins PD, Niedernhofer LJ, Kang J (2010). Accelerated aging of intervertebral discs in a mouse model of progeria. *J Orthop Res*. **28**, 1600-1607.

Wang H, Kirkland JL, Hollenberg CH (1989). Varying capacities for replication of rat adipocyte precursor clones and adipose tissue growth. *J. Clin. Invest.* **83**, 1741-1746.

Wang S, Xu M, Li F, Wang X, Bower KA, Frank JA, Lu Y, Chen G, Zhang Z, Ke Z, Shi X, Luo J (2012). Ethanol promotes mammary tumor growth and angiogenesis: the involvement of chemoattractant factor MCP-1. *Breast Cancer Res Treat*. **133**, 1037-1048.
