# Supplementary material for: The Achilles’ heel of senescent cells: from transcriptome to senolytic drugs
Source: Aging Cell. 2015 Apr 22;14(4):644–58. doi: 10.1111/acel.12344 (PMC4531078; doi:10.1111/acel.12344)
Supplement: Supplementary file 1 [file acel0014-0644-sd1.zip › supplementalfigurelegends.docx]

**Supplemental Information**

**Supplemental Fig 1**: The extent of mRNA knock-down by the various siRNAs (**A**-**B**) and siRNA transfection (**C1-10**) were equivalent in senescent preadipocytes (**C5,6**), proliferating non-senescent preadipocytes (**C1,2**), differentiated non-senescent preadipocytes (**C3,4**), proliferating HUVECs (**C7,8)**, and senescent HUVECs (**C9,10**). Cells were transfected with siRNAs or a siGLO™ Red Transfection Indicator (**C1,3,5,7,9**). **C2,4,6,8,10** show phase contrast images to illustrate cell density. After 48 hours, cells were harvested and mRNA was quantified by RT-PCR. Histograms represent the mean±SEM for 3 independent replicates

**Supplemental Fig 2:** Validation of gene expression data **(A-B**) Confirmation of the siRNA data using a second siRNA to knock-down expression against each of the indicated genes in radiation-induced senescent *vs*. non-senescent human subcutaneous preadipocytes and HUVECs. **(C**) Reducing expression of EFNB2 or a PI3K isoform, PI3KCG, distinct from PI3KCD, had no effect on the viability of either senescent or non-senescent preadipocytes. **(D**) Senescent cells can be selectively targeted by suppressing expression of pro-survival mechanisms using RNA interference in serially-passaged human preadipocytes. Targeting survival pathways by siRNA reduces viability (ATPLite) of serial passage-induced senescent human abdominal subcutaneous primary preadipocytes to a greater extent than non-senescent proliferating cells. **P*<0.05, N=4, T test. Primary preadipocytes were serially passaged for 28 population doublings. Senescence was confirmed by SA-βGal assay (75-85%). **(E**) Senolytic siRNAs did not interfere with the viability of quiescent, differentiated preadipocytes. Confluent human abdominal subcutaneous 4^th^ passage primary preadipocytes were treated with differentiation-inducing medium for 20 days and then cells were transfected with each siRNA using RNAiMAX reagent and maintained in the maintenance media for 2 days. Media were changed back to regular culture media for 5 days. Cell viability was assayed by ATPLite on the 5^th^ day. **(F**) The expression of EFNB1, EFNB3, PI3KCD, and p21 proteins was reduced to a similar extent by siRNA in senescent and non-senescent preadipocytes. Human abdominal subcutaneous 4^th^ passage primary preadipocytes were transfected with EFNB1, EFNB3, PI3KCD, p21, or scrambled siRNAs. After 48 hours, cells were harvested and protein was assayed by Western immunoanalysis. β-actin was assayed as a loading control.

Note that although many anti-apoptotic regulatory pathways were up-regulated in senescent cells by array pathway analysis, not every individual anti-apoptotic regulator was increased. We speculated that the ones that are less highly expressed, including the ephrins and PI3KCD, may be good senolytic targets because by down-regulating them to the same extent in senescent and non-senescent cells we could selectively elicit a functional change (apoptosis) in senescent cells without affecting non-senescent cells. If instead we targeted the most highly expressed anti-apoptotic factors, this would require a greater extent of knock-down to induce apoptosis and is more apt to elicit a non-specific effect in all cells.

**Supplemental Fig 3**: **(A)** PAI-1 siRNA decreases ATPLite in senescent preadipocytes. This could not be confirmed using a 2nd siRNA. No effect was observed in HUVECs. **P*<0.05, N=6, T test. (**B**) PAI-1 antagonists are in clinical trials for inducing apoptosis in gastrointestinal and xenografted lung and skin cancers ([Mutoh *et al.* 2008](#_ENREF_2); [Masuda *et al.* 2013](#_ENREF_1)). The PAI-1 inhibitor tiplaxtinin exhibited possible senolytic effects at a high concentration, 200 mM, in preadipocytes assayed by ATPLite. N=2 subjects; means±SEM are shown.

**Supplemental Fig 4:** Dasatinib is more senolytic than imatinib. Human subcutaneous preadipocytes were exposed to 10 Gy radiation 25 days before or were non-irradiated. Cells were plated at sub-confluent (50%) density at day 0 (=100%). After 3 days of exposure to dasatinib (**A**) or imatinib (**B**), ATPLite was used to assay viable cells. Note that by day 3, the proliferating cells had increased to ~190% of plated cells at day 0, while senescent cells did not divide, remaining near 100%. The distance between the red lines (denoting senescent cells plated) and the data points corresponds to % senescent cells eliminated. Dasatinib caused a dose-dependent decrease in senescent cells to ~50% of those plated, up to the concentration at which it also inhibited non-senescent cell viability (1000 nM), whereas imatinib did not reduce senescent cell viability more than that of proliferating cells at any concentration. Note that imatinib EC_50_ against cancer cells is ~10 fold higher than dasatinib ([O'Hare *et al.* 2005](#_ENREF_3)). N=4 independent experiments, means±SEM are shown.

**Supplemental Fig 5: (A**) Over 95 percent of radiated cultured human preadipocytes are SA-βGal^+^. **A1**: Proliferating 4^th^ passage primary human abdominal subcutaneous preadipocytes assayed for SA-βGal (male, 57 years old, BMI 29.5). **A2**: Fourth passage primary human abdominal subcutaneous preadipocytes 25 days following 10 Gy x-ray radiation when they were assayed for SA-βGal. Representative of 3 experiments. **A3**: Fifth passage proliferating HUVECs assayed for SA-βGal. **A4**: Fifth passage HUVECs 10 days following 10 Gy x-ray radiation when they were assayed for SA-βGal. **(B**) % SA-βGal^+^ cells in cultured non-senescent proliferating *vs*. radiated *vs*. serial passage-induced senescent preadipocytes. **(C**) % SA-βGal^+^ cells in cultured non-senescent proliferating *vs*. radiation-induced senescent HUVECs.

**Supplemental Fig 6**: Elimination of senescent *vs*. non-senescent human preadipocytes and HUVECs by siRNA knockdown. **A**: Abdominal subcutaneous primary human preadipocytes were transfected with EFNB-1, EFNB-3, PI3KCD, p21, PAI-2, or scrambled siRNAs. The siRNAs caused death selectively of senescent *vs*. non-senescent preadipocytes. Representative of 5 experiments. Figures **A1-6** are senescent preadipocytes; **A7-12** are non-senescent, proliferating 4^th^ passage preadipocytes. **B**: Radiation-induced senescent HUVECs **(B1-3)** and non-senescent, proliferating HUVECs **(B4-6)** were transfected with EFNB-1, BCL-xL, or scrambled siRNAs. After 5 days, cultures were stained with crystal violet.

**Supplemental Fig 7**: D and Q cause apoptosis in senescent primary human preadipocytes. **A**: DNA fragmented substantially in the nucleus of senescent cells after a 24-48 hour exposure to D+Q (DNA fragmentation circled in red). **B**: Terminal deoxynucleotidyl transferase dUTP nick end labeling (TUNEL) with florescent green dye shows apoptotic cells.

**Supplemental Fig 8**: Leading edges of gene sets at higher resolution. Heat maps of the leading edges of gene sets related to anti-apoptotic function, “negative regulation of apoptosis” (**A**) and “anti-apoptosis” (**B**), in senescent *vs*. non-senescent preadipocytes are shown (red = higher; blue = lower). Samples are ordered from left to right first by proliferative state and then by subject (N=8), and the rows are ordered from top to bottom by the absolute value of the Student t statistic computed between the senescent and proliferating cells (*i.e*., from greatest to least significance). Each column represents one subject.

**Supplemental Fig 9**: Periodic or acute treatment of *Ercc1^-/Δ^* mice with D+Q attenuates senescence in tissues of *Ercc1^-/Δ^* mice. **A**: Liver sections of mice from 4 mice, 2 treated weekly with D+Q and 2 that received vehicle only (V) stained for SA-βGal (5X magnification). **B**: Quantitation of the number of SA-βGal^+^ cells in ten random fields of each section. *p=0.032, Student’s t test. **C**: SA-βGal staining of inguinal fat from 4 mice, 2 treated acutely with D+Q (3 doses in one week M,W,F) and 2 that received vehicle only. Interestingly, more senescence was detected in male *Ercc1^-/Δ^* mice, affording greater differences in male mice treated with D+Q than female mice (see Supplemental Fig. 12).

**Supplemental Fig. 10**: Representative images of *Ercc1^-/Δ^* mice from the D+Q treatment group or vehicle only. Splayed feet are an indication of dystonia and ataxia. Images of 15 week old *Ercc1^-/∆^* mice (**A-D**). Aligned left and right are age-matched animals treated periodically with vehicle only or D+Q. Signs of aging and frailty that appear improved in the D+Q group are indicated with arrows. Images of 16 week old *Ercc1^-/Δ^* mice (**E-H**). Aligned left and right are sibling pairs treated periodically with vehicle only or D+Q. Signs of aging and frailty that appear improved in the D+Q group are indicated with arrows.

**Supplemental Fig. 11**: Pairwise analysis of the age at onset of all age-related symptoms measured in sibling pairs of *Ercc1^-/Δ^* mice. Each color represents a different symptom. The height of the bar indicates the severity of the symptom at a particular age. The composite height of the bar is an indication of the animals overall health (lower=better health). Many symptoms are delayed in onset in the *Ercc1^-/Δ^* mice treated with D+Q compared to siblings treated with vehicle only, for example ataxia (orange), grip strength (red), hind limb paralysis (green), and gait disorder (dark blue). Many symptoms are also attenuated, for example dystonia (light blue), gait disorders (dark blue), and ataxia (orange).

**Supplemental Fig. 12**: Differences in the response of male and female *Ercc1^-/Δ^* mice to D+Q. **A**: Male *Ercc1^-/Δ^* mice have higher expression of the senescence marker p16 mRNA in liver lysates. Therefore the impact of D+Q on p16 expression is more dramatic in male mice. **B**: Glycosaminoglycan expression is significantly increased in the intervertebral disc nucleus pulposa of male *Ercc1^-/Δ^* mice. Sexual dimorphisms need to be investigated in the future with larger cohorts of mice. *p<0.03, Student’s t test.

**Supplemental Fig. 13**: Representative images of the tissue sections scored for age-related histopathological changes. Both sections are from a female mouse treated weekly with 5 mg/kg dasatinib and 50 mg/kg quercetin for 10 weeks then euthanized at 16 weeks of age. Shown are hematoxylin and eosin stained sections of the liver and kidney imaged at 200X. Hepatopathy: the liver shows a combination of degenerative changes, including sporadic necrosis, regenerative changes, and markedly distended hepatocytes with glycogen and with or without discrete membrane-bound lipid inclusions. Nephropathy: the kidney shows a combination of minor sclerotic changes in a glomerulus, mild inflammation with cell infiltration, and anisonucleosis in the renal tubular epithelium.

**Supplemental Table 1.** siRNAs screened for senolytic activity. 39 siRNAs were screened by ATPLite assay for effects on senescent and non-senescent preadipocyte and HUVEC viability.

**Supplemental Table 2**: Human and mouse TagMan primer probes used.

**Supplemental Table 3**: Effects of senolytic treatment (D+Q) on cardiac function.

**References**

Masuda T, Hattori N, Senoo T, Akita S, Ishikawa N, Fujitaka K, Haruta Y, Murai H, Kohno N (2013). SK-216, an inhibitor of plasminogen activator inhibitor-1, limits tumor progression and angiogenesis. Mol Cancer Ther. 12, 2378-2388.

Mutoh M, Niho N, Komiya M, Takahashi M, Ohtsubo R, Nakatogawa K, Ueda K, Sugimura T, Wakabayashi K (2008). Plasminogen activator inhibitor-1 (Pai-1) blockers suppress intestinal polyp formation in Min mice. Carcinogenesis. 29, 824-829.

O'Hare T, Walters DK, Stoffregen EP, Jia T, Manley PW, Mestan J, Cowan-Jacob SW, Lee FY, Heinrich MC, Deininger MW, Druker BJ (2005). In vitro activity of Bcr-Abl inhibitors AMN107 and BMS-354825 against clinically relevant imatinib-resistant Abl kinase domain mutants. *Cancer Res*. **65**, 4500-4505.
