# Supplementary material for: The Achilles’ heel of senescent cells: from transcriptome to senolytic drugs
Source: Aging Cell. 2015 Apr 22;14(4):644–58. doi: 10.1111/acel.12344 (PMC4531078; doi:10.1111/acel.12344)
Supplement: Supplementary file 1 [file acel0014-0644-sd1.zip › Supplemental Table 3.docx]

**Supplemental Table 3. Effects of senolytic treatment (D+Q) on cardiac function**

Echocardiography was used to provide measurements of systolic and diastolic cardiac function in anesthetized mice. Note that senolytic treatment with D + Q improves fractional shorting while leaving measures of diastolic function (E/e’), general cardiac function (MPI, a measure that combines systolic and diastolic function), and left ventricular mass unchanged. Asterisks denote *P*< 0.05.

|  | **Systolic Function** | **Diastolic Function** | | | **General Cardiac Function** | **Left Ventricular Mass** | | |
| --- | --- | --- | --- | --- | --- | --- | --- | --- |
|  | **FS**  **(%)** | **E**  **(mm/sec)** | **e’**  **(mm/sec)** | **E/e’** | **MPI** | **LVMe**  **(mg)** | **Body weight**  **(g)** | **LVMe/BW**  **(mg/g)** |
| **Vehicle** | 41 ± 4 | 665 ± 35 | 22 ± 2 | 30 ± 3 | 0.77 ± 0.03 | 112 ± 5 | 31.3 ± 0.9 | 3.6 ± 0.2 |
| **D + Q** | 46 ± 3* | 578 ± 43 | 20 ± 2 | 30 ± 2 | 0.72 ± 0.03 | 107 ± 7 | 30.9 ± 0.8 | 3.5 ± 0.2 |

FS = Fractional Shortening, LVMe = echocardiographic estimate of left ventricular mass, BW = body weight, E = tissue Doppler measurement of mitral peak velocity of early filling, e’ = tissue Doppler measurement of diastolic mitral annular velocity, MPI = myocardial performance index.
