# Supplementary material for: The Achilles’ heel of senescent cells: from transcriptome to senolytic drugs
Source: Aging Cell. 2015 Apr 22;14(4):644–58. doi: 10.1111/acel.12344 (PMC4531078; doi:10.1111/acel.12344)
Supplement: Supplementary file 1 [file acel0014-0644-sd1.zip › Supplemental Table 2 TagManPrimer Assays.docx]

**Supplemental Table 2: List of Human and Mouse TagMan Primer Probe Assays**

| **Gene Symbol** | **Assay ID** |  | **Gene Symbol** | **Assay ID** |
| --- | --- | --- | --- | --- |
| ABL1 | Hs01104728_m1 |  | CDKN1A | Hs00355782_m1 |
| ABL2 | Hs00943652_m1 |  | KDR, VEGFR2 | Hs00911700_m1 |
| HIF1A | Hs00153153_m1 |  | FLT1, VEGFR1 | Hs01052961_m1 |
| BCL2L1 | Hs00236329_m1 |  | KIT | Hs00174029_m1 |
| MCL1 | Hs01050896_m1 |  | SERPINE1, PAI-1 | Hs01126606_m1 |
| BST1 | Hs00174709_m1 |  | PAI-2 | Hs01010736_m1 |
| PGAP1 | Hs01088726_m1 |  | PPM1A | Hs01056778_g1 |
| CD38 | Hs01120071_m1 |  | PPM1B | Hs00952176_m1 |
| FYN | Hs00941600_m1 |  | PTK2 | Hs01056457_m1 |
| CHEK1 | Hs00967506_m1 |  | PDGFB | Hs00966522_m1 |
| SRC | Hs01082246_m1 |  | PDGFA | Hs00964426_m1 |
| EEF2K | Hs00179434_m1 |  | BCL2 | Hs00608023_m1 |
| EFNA3 | Hs00191913_m1 |  | PPM1L | Hs00289330_m1 |
| EFNB3 | Hs00154861_m1 |  | PIK3CA | Hs00907957_m1 |
| EEF2 | Hs00157330_m1 |  | PIK3CB | Hs00927728_m1 |
| GSK3B | Hs01047719_m1 |  | PIK3CD | Hs00192399_m1 |
| MKRN1 | Hs01570537_g1 |  | PIK3CG | Hs00932389_g1 |
| EFNB1 | Hs00270004_m1 |  | PRKCB | Hs00176998_m1 |
| EFNB2 | Hs00187950_m1 |  | TBP | Hs00427620_m1 |
| EPHA4 | Hs00177874_m1 |  | TBP | Mm00446971_m1 |
| EPHA2 | Hs00171656_m1 |  | Cdkn2a | Mm00494449_m1 |
| MCL1 | Hs01050896_m1 |  | Cdkn1a | Mm04205640_g1 |
| CDKN1A | Hs00355782_m1 |  | TBP | Mm00446971_m1 |
