# Supplementary material for: The Achilles’ heel of senescent cells: from transcriptome to senolytic drugs
Source: Aging Cell. 2015 Apr 22;14(4):644–58. doi: 10.1111/acel.12344 (PMC4531078; doi:10.1111/acel.12344)
Supplement: Supplementary file 1 [file acel0014-0644-sd1.zip › Supplemental Table 1.docx]

**Supplemental Table 1: Pro-survival genes selected for expression interference using siRNA**

**1A.** siRNA were transduced into duplicate cultures of primary human abdominal preadipocytes and HUVEC cells +/- 10 Gy ionizing radiation to induce cellular senescence. Senescence was confirmed by SA-βGal. Viability of senescent and non-senescent cells was determined by ATPLite assay. Listed is the percent decrease in viability 3 days post-transduction of the cells with each siRNA. ↓ indicates decreased viability. nd indicates not determined.

|  |  | **Preadipocytes** | | **HUVECs** | |  |
| --- | --- | --- | --- | --- | --- | --- |
| **siRNA** |  | **Proliferating** | **Senescent** | **Proliferating** | **Senescent** |  |
| ABL1 |  | 0% | 0% | 0% | 0% |  |
| ABL2 |  | 0% | 0% | 0% | 0% |  |
| ABL1+ ABL2 |  | 0% | 0% | 0% | 0% |  |
| Src |  | mild cytostasis | 10-20% | 0% | 0% |  |
| AKT1+AKT2 |  | ↓ | ↓ | ↓ | ↓ |  |
| EEF2 |  | ↓ | ↓ | 0% | 5-10% |  |
| EFNB1 |  | 0% | 30-40% | nd | nd |  |
| EFNB2 |  | 0% | 0% | nd | nd |  |
| EFNB3 |  | 0% | 30-40% | nd | nd |  |
| GSK3β |  | 0% | 10% | 10% | 40% |  |
| HIF-1α |  | 0% | 30% | 30% | 40% |  |
| PDGFA |  | 0% | 0% | 0% | 0% |  |
| PDGFB |  | nd | nd | 30-40% | 30-40% |  |
| PKCβ1 |  | 0% | 0% | nd | nd |  |
| PTK2 |  | 0% | 0% | 0% | 10-20% |  |
| CHEK1 |  | 0% | 10% | 0% | nd |  |
| PAI1 |  | 0% | 35% | 0% | 0% |  |
| PAI2 |  | 10-20% | 30-40% | 10-20% | 0% |  |
| PI3KCB |  | 0% | 0% | nd | nd |  |
| PI3K CD |  | 0% | 40-50% | 30-40% | 40-50% |  |
| PI3K CG |  | 0% | 0% | nd | nd |  |
| PI3KCA |  | 0% | 0% | nd | nd |  |
| PPM1A |  | 0% | 0% | 0% | 0% |  |
| PPM1B |  | 0% | 0% | 20% | 30% |  |
| PPM1L |  | 0% | 0% | 0% | 0% |  |
| BCL2 |  | nd | nd | 50% | 50% |  |
| BCL-xL |  | 30-40% | 30-40% | 30-40% | 70-80% |  |
| MCL1 |  | 10-20% | 10-20% | 30-40% | 30-40% |  |
| BST1 |  | 0% | 10-20% | nd | nd |  |
| Bim |  | 0% | 0% | nd | nd |  |
| CD38 |  | 0% | 0% | nd | nd |  |
| p21 |  | 0% | 20-30% | 20-30% | 20-30% |  |
| EPHA2 |  | 0% | 0% | 0% | nd |  |
| EPHA4 |  | 0% | 0% | 0% | nd |  |

1B. Same as above except viability was determined by brightfield imaging of cells.

|  | **Preadipocytes** | | **HUVECs** | |
| --- | --- | --- | --- | --- |
| **siRNA** | **Non-senescent** | **Senescent** | **Non-senescent** | **Senescent** |
| EFNA3 | 0% | 0% | nd | nd |
| MKRN1 | 0% | 0% | 30-40% | 40-50% |
| FYN | 0% | 0% | nd | nd |
| c-Kit | 0% | 0% | nd | nd |
| VEGFR1 | 0% | 0% | nd | nd |
| VEGFR2 | 0% | 0% | nd | nd |
