# Supplementary material for: The Achilles’ heel of senescent cells: from transcriptome to senolytic drugs
Source: Aging Cell. 2015 Apr 22;14(4):644–58. doi: 10.1111/acel.12344 (PMC4531078; doi:10.1111/acel.12344)

SUPPLEMENTAL FIGURE 10

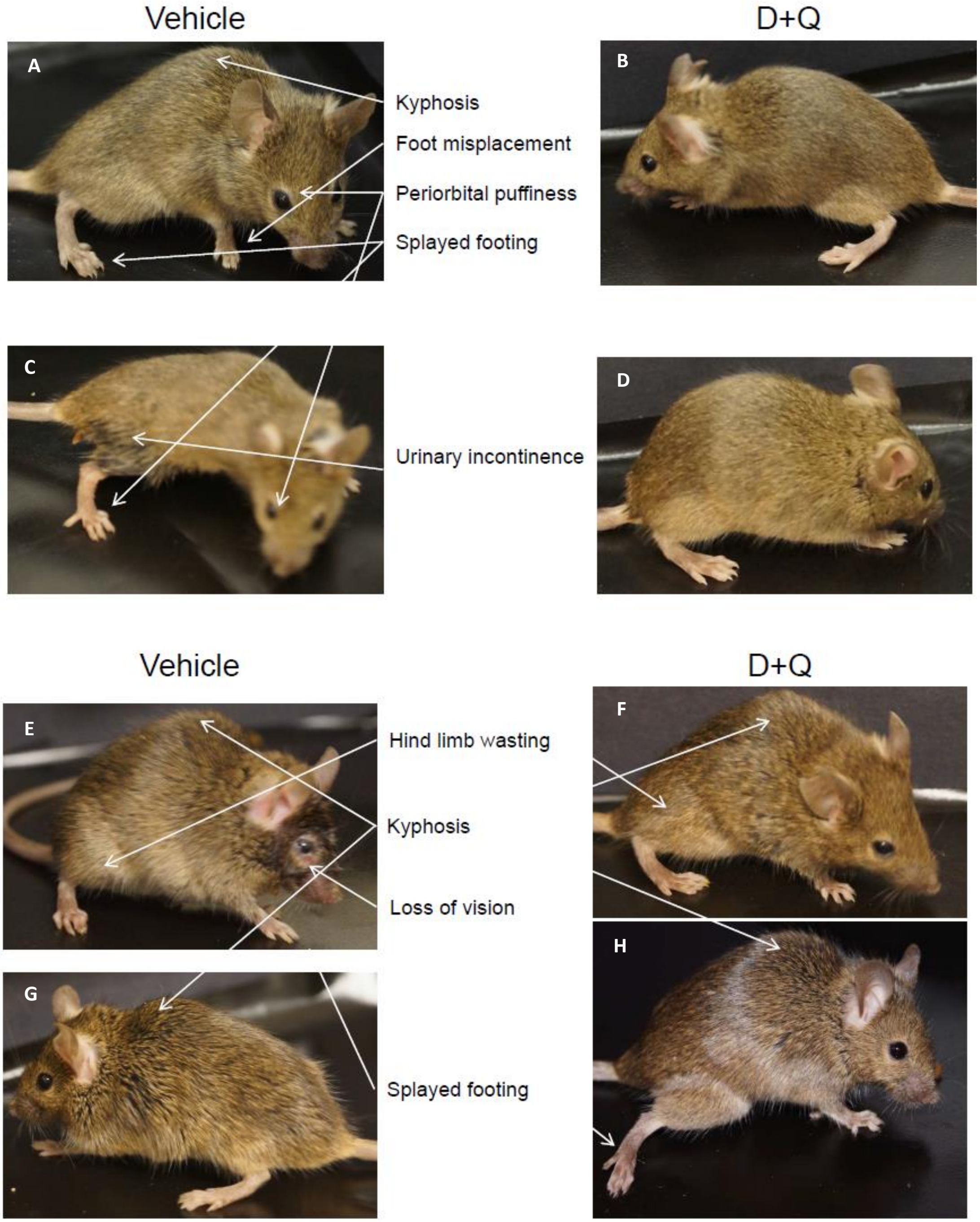

Supplement: Supplementary file 1 [file acel0014-0644-sd1.zip › supplemental Figure 10.pdf]
