# Supplementary figures and images for: The Achilles’ heel of senescent cells: from transcriptome to senolytic drugs
Source: Aging Cell. 2015 Apr 22;14(4):644–58. doi: 10.1111/acel.12344 (PMC4531078; doi:10.1111/acel.12344)

SUPPLEMENTAL FIGURE 13

Liver

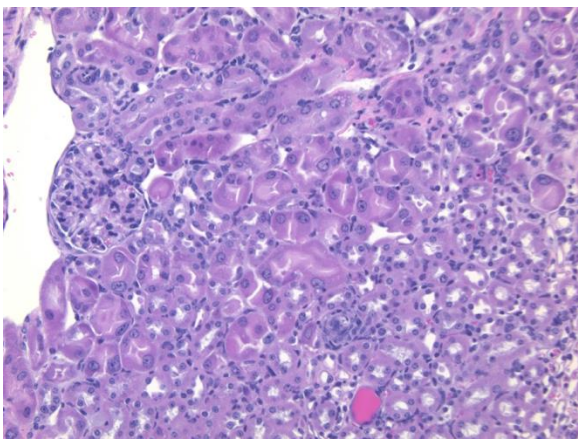

Kidney

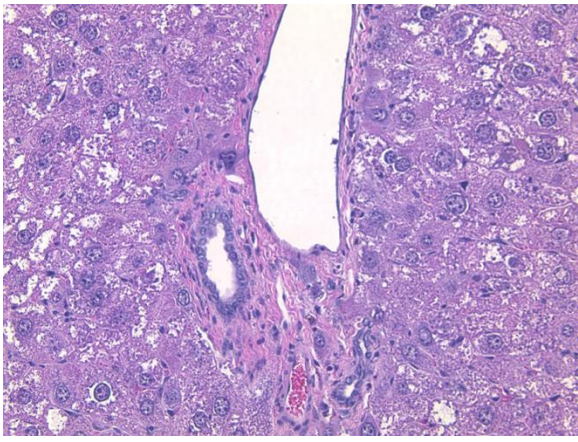

Supplement: Supplementary file 1 [file acel0014-0644-sd1.zip › Supplemental Figure 13.pdf]

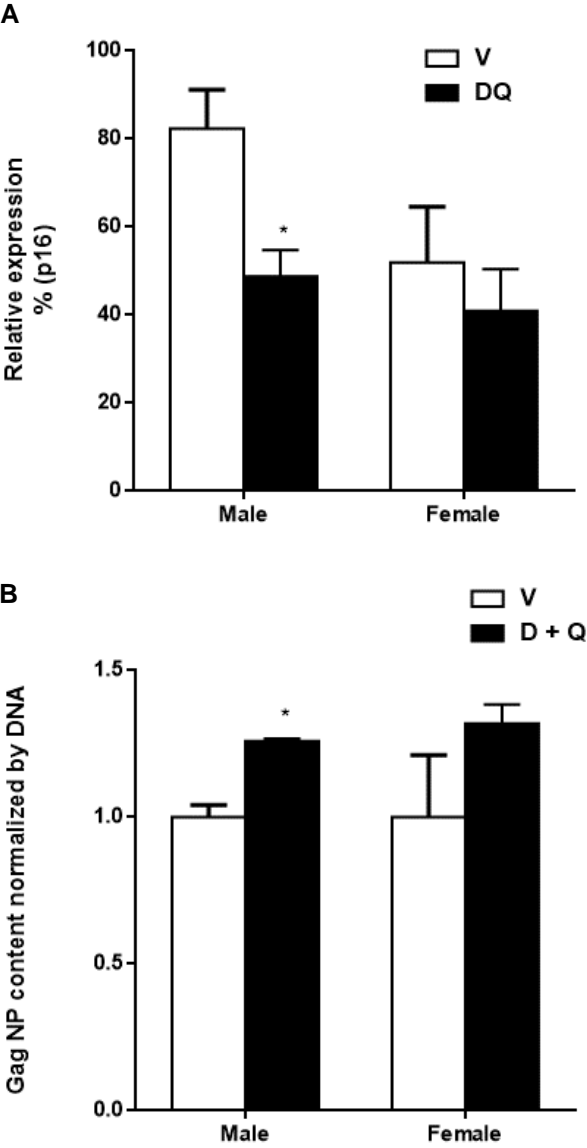

Supplement: Supplementary file 1 [file acel0014-0644-sd1.zip › Supplemental Figure 12.pdf]

SUPPLEMENTAL FIGURE 11

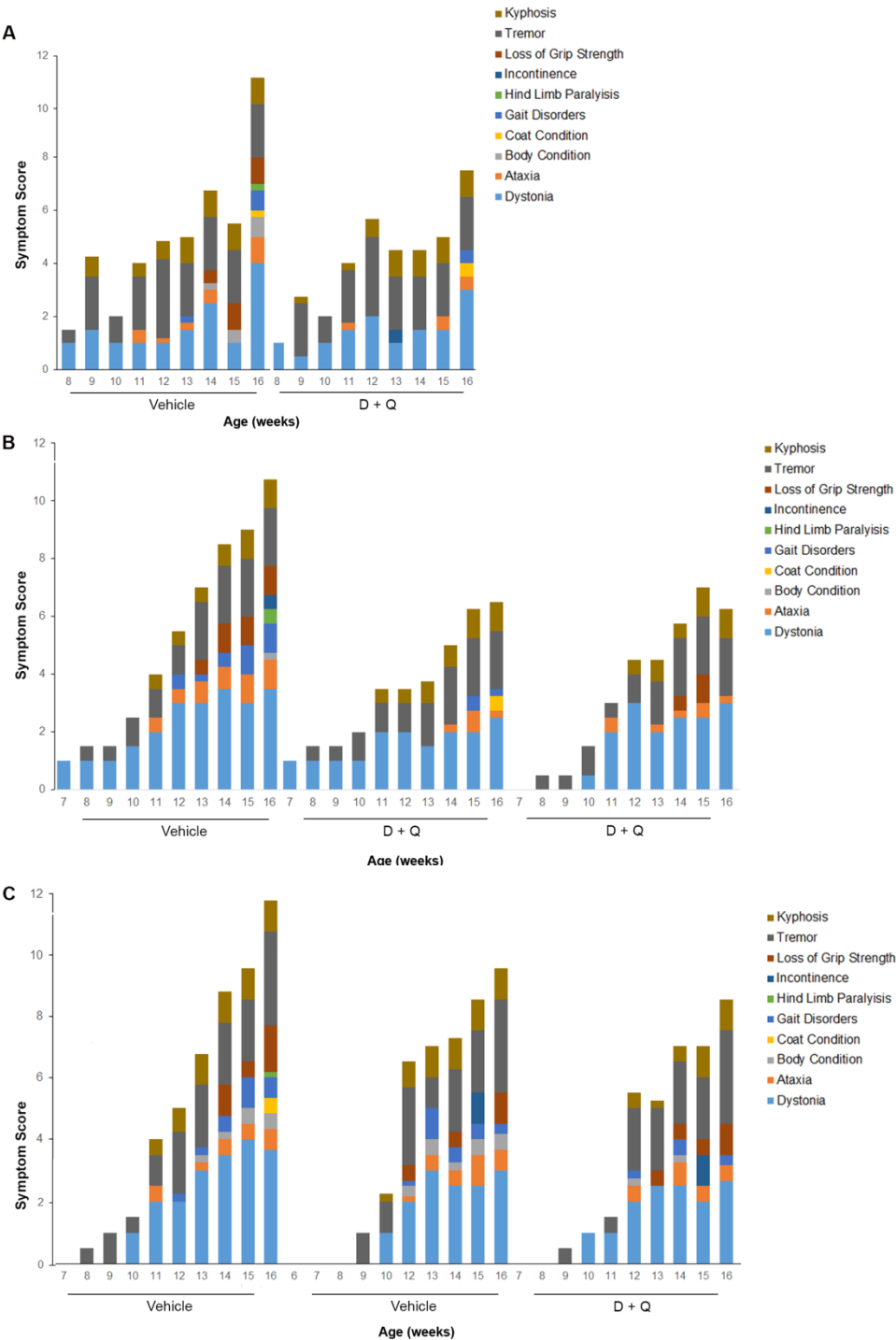

Supplement: Supplementary file 1 [file acel0014-0644-sd1.zip › Supplemental Figure 11.pdf]

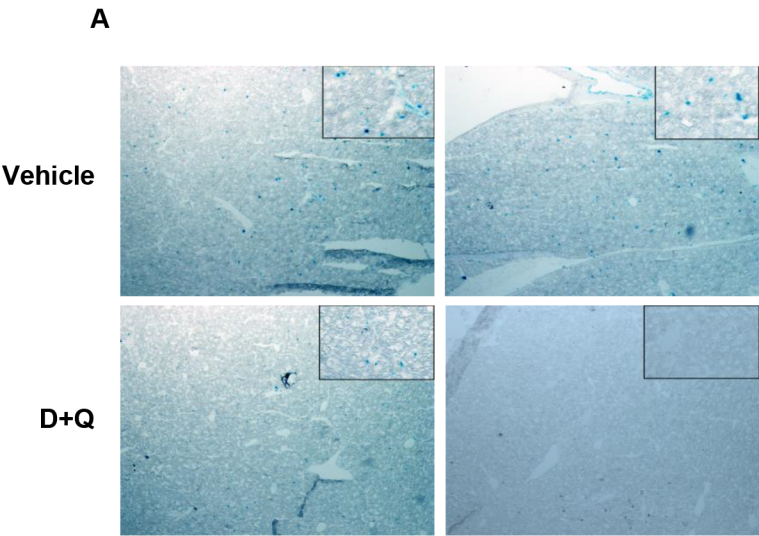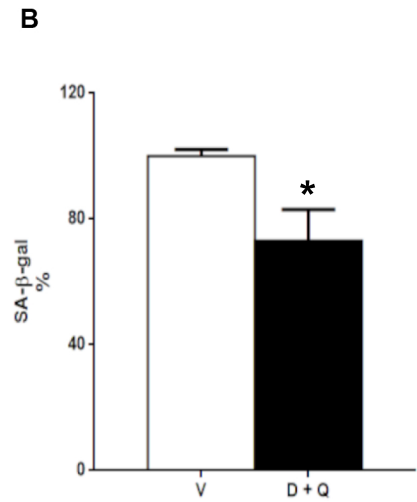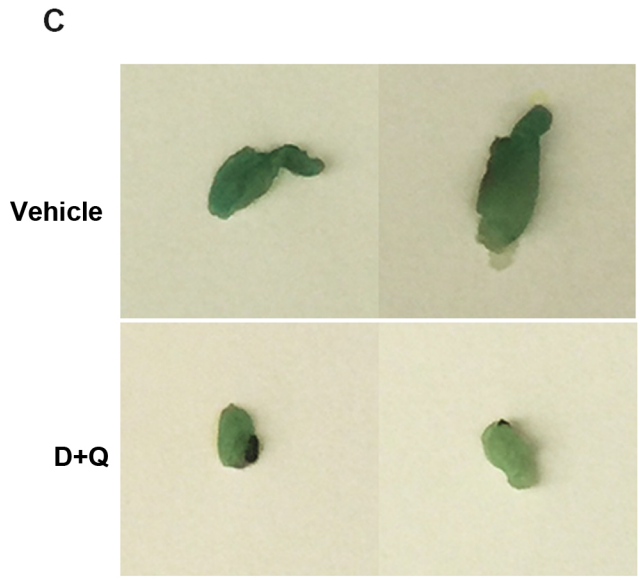

Supplement: Supplementary file 1 [file acel0014-0644-sd1.zip › Supplemental Figure 9.pdf]

SUPPLEMENTAL FIGURE 8

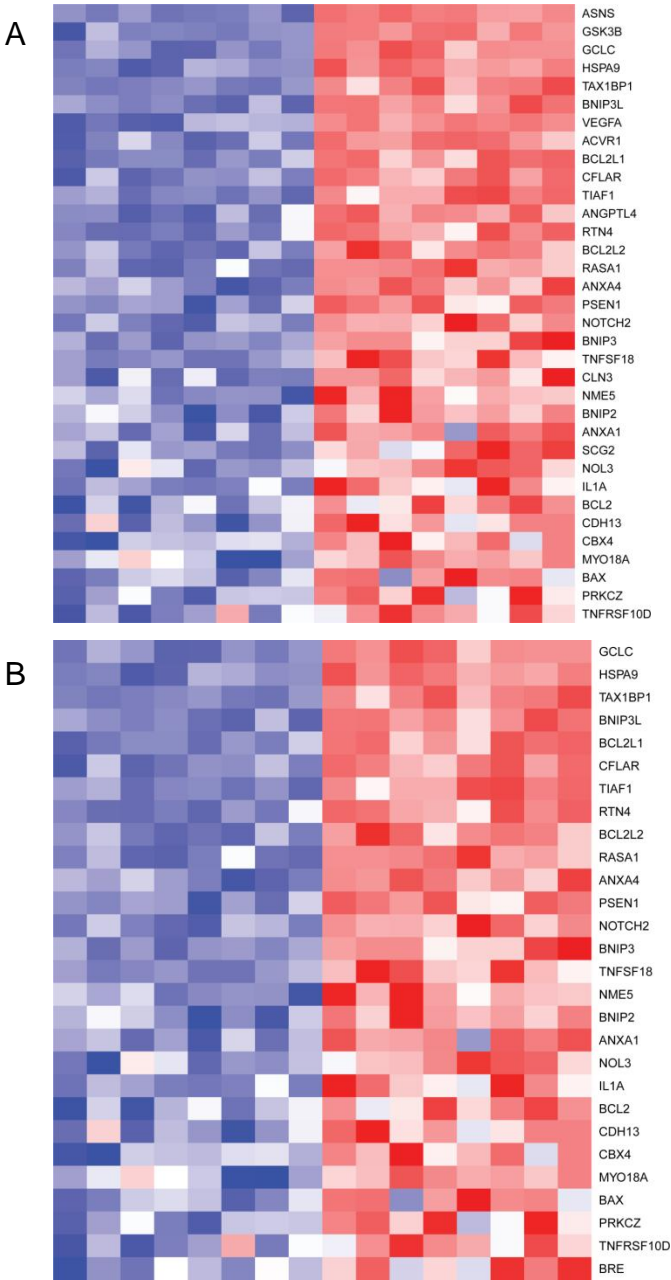

Supplement: Supplementary file 1 [file acel0014-0644-sd1.zip › Supplemental Figure 8.pdf]

SUPPLEMENTAL FIGURE 7

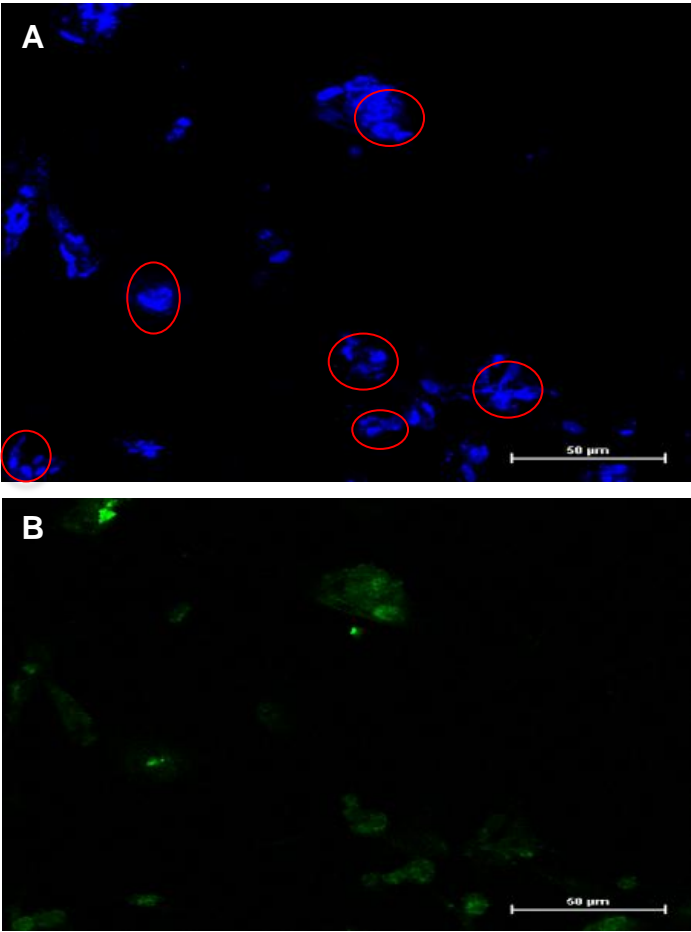

Supplement: Supplementary file 1 [file acel0014-0644-sd1.zip › Supplemental Figure 7.pdf]

SUPPLEMENTAL FIGURE 6

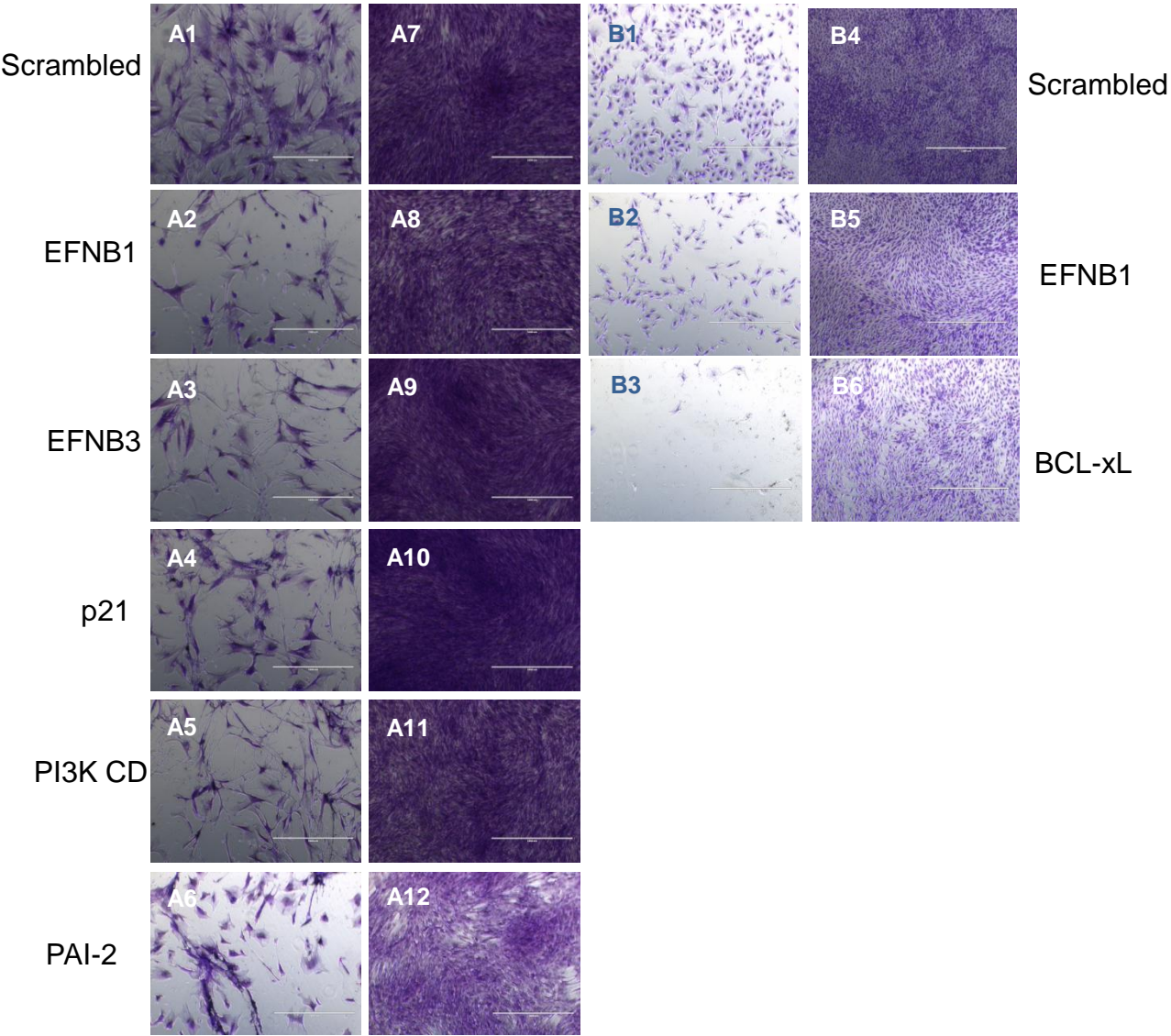

Supplement: Supplementary file 1 [file acel0014-0644-sd1.zip › Supplemental Figure 6.pdf]

SUPPLEMENTAL FIGURE 5

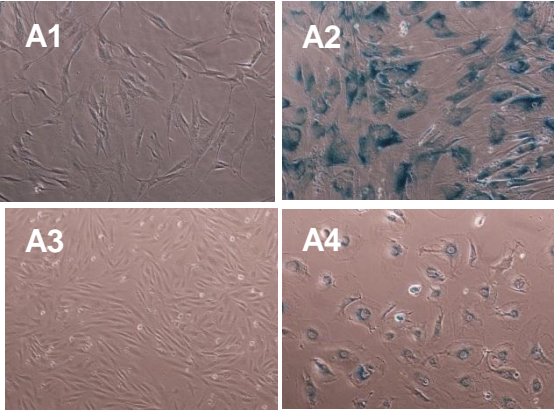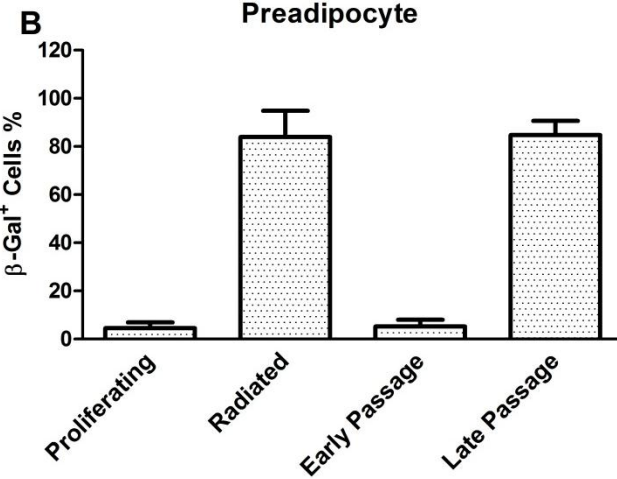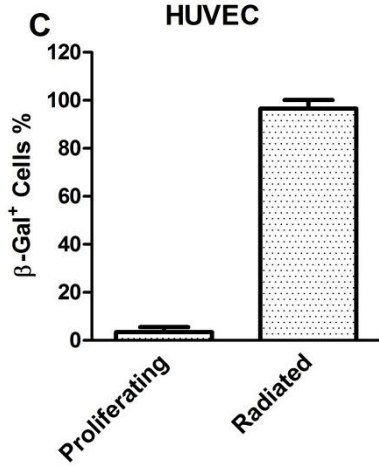

Supplement: Supplementary file 1 [file acel0014-0644-sd1.zip › Supplemental Figure 5.pdf]

SUPPLEMENTAL FIGURE 4

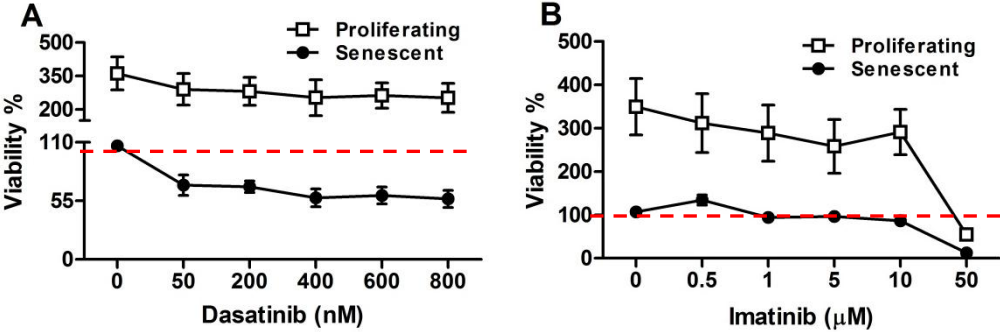

Supplement: Supplementary file 1 [file acel0014-0644-sd1.zip › Supplemental Figure 4.pdf]

SUPPLEMENTAL FIGURE 3

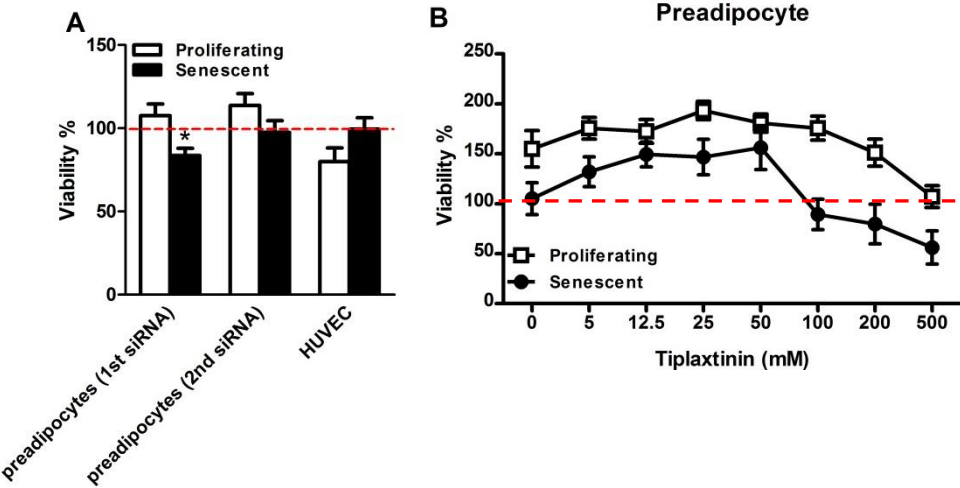

Supplement: Supplementary file 1 [file acel0014-0644-sd1.zip › Supplemental Figure 3.pdf]

SUPPLEMENTAL FIGURE 2

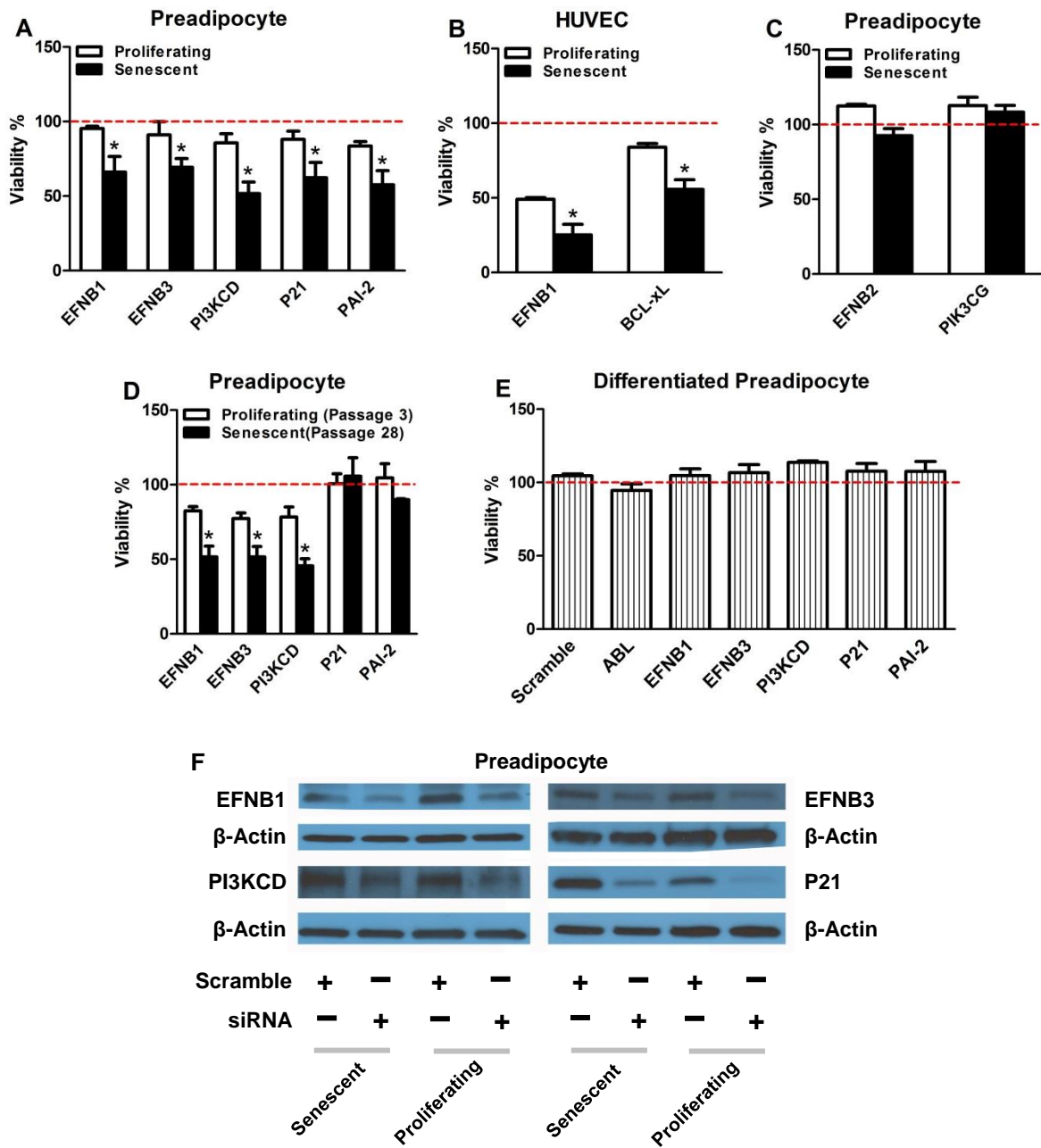

Supplement: Supplementary file 1 [file acel0014-0644-sd1.zip › Supplemental Figure 2.pdf]

SUPPLEMENTAL FIGURE 1

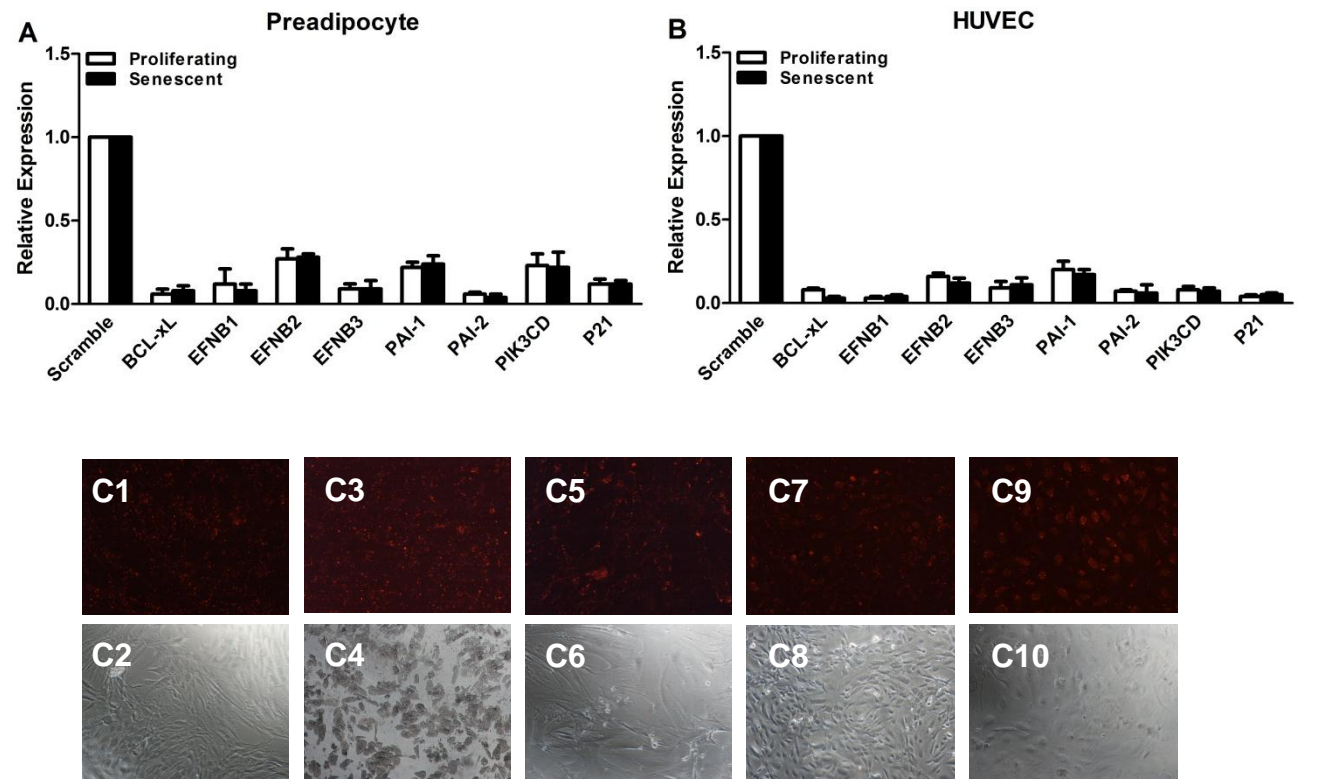

Supplement: Supplementary file 1 [file acel0014-0644-sd1.zip › Supplemental Figure 1.pdf]
